# Japan society of clinical oncology position paper on appropriate clinical use of molecular residual disease (MRD) testing

**DOI:** 10.1007/s10147-024-02683-0

**Published:** 2025-02-07

**Authors:** Shin Kobayashi, Yoshiaki Nakamura, Tadayoshi Hashimoto, Hideaki Bando, Eiji Oki, Takahiro Karasaki, Hidehito Horinouchi, Yukinori Ozaki, Hiroji Iwata, Taigo Kato, Hideaki Miyake, Akihiro Ohba, Masafumi Ikeda, Tatsuyuki Chiyoda, Kosei Hasegawa, Takao Fujisawa, Kazuto Matsuura, Kenjiro Namikawa, Shugo Yajima, Takayuki Yoshino, Kiyoshi Hasegawa

**Affiliations:** 1 Department of Hepatobiliary and Pancreatic Surgery, National Cancer Center Hospital East, 6-5-1, Kashiwanoha, Kashiwa-shi, Chiba 277-8577 Japan; 2 Perioperative Treatment Development Promotion Office, National Cancer Center Hospital East, 6-5-1, Kashiwanoha, Kashiwa-shi, Chiba 277-8577 Japan; 3 Department of Gastrointestinal Oncology, National Cancer Center Hospital East, 6-5-1, Kashiwanoha, Kashiwa-shi, Chiba 277-8577 Japan; 4 Translational Research Support Office, National Cancer Center Hospital East, 6-5-1, Kashiwanoha, Kashiwa-shi, Chiba 277-8577 Japan; 5 International Research Promotion Office, National Cancer Center Hospital East, 6-5-1, Kashiwanoha, Kashiwa-shi, Chiba 277-8577 Japan; 6 Department of Surgery and Science, Graduate School of Medical Sciences, Kyushu University, Fukuoka, Japan; 7 Department of Thoracic Surgery, Respiratory Center, Toranomon Hospital, Tokyo, Japan; 8 Department of Thoracic Oncology, National Cancer Center Hospital, Tokyo, Japan; 9 Department of Breast Medical Oncology, Cancer Institute Hospital of Japanese Foundation for Cancer Research, Tokyo, Japan; 10 Department of Advanced Clinical Research and Development, Nagoya City University Graduate School of Medical Sciences, Nagoya, Japan; 11 Department of Urology, Osaka University Graduate School of Medicine, Suita, Japan; 12 Division of Urology, Kobe University Graduate School of Medicine, Kobe, Japan; 13 Division of Gastrointestinal Oncology, Shizuoka Cancer Center, Nagaizumi, Japan; 14 Department of Hepatobiliary and Pancreatic Oncology, National Cancer Center Hospital East, Kashiwa, Japan; 15 Department of Obstetrics and Gynecology, Keio University School of Medicine, Tokyo, Japan; 16 Department of Gynecologic Oncology, Saitama Medical University International Medical Center, Hidaka, Saitama Japan; 17 Department of Head and Neck Medical Oncology, National Cancer Center Hospital East, Kashiwa, Japan; 18 Department of Head and Neck Surgery, National Cancer Center Hospital East, Kashiwa, Japan; 19 Department of Dermatologic Oncology, National Cancer Center Hospital, Tokyo, Japan; 20 Department of Urology, National Cancer Center Hospital East, Kashiwa, Japan; 21 Kindai University Faculty of Medicine, Osakasayama, Japan; 22 Department of Surgery, Graduate School of Medicine, Hepato-Biliary-Pancreatic Surgery Division, The University of Tokyo, Tokyo, Japan

**Keywords:** Circulating tumor DNA, Molecular residual disease, Solid tumor

## Abstract

Although the 5-year relative survival rates for resectable solid tumors have improved over the past few years, the risk of postoperative recurrence necessitates effective monitoring strategies. Recent advancements in molecular residual disease (MRD) testing based on circulating tumor DNA (ctDNA) analysis have shown considerable promise in the context of predicting recurrence; however, significant barriers to widespread clinical implementation remain—mainly, low awareness among healthcare professionals, high costs, and lack of standardized assays and comprehensive evidence. This position paper, led by the Japan Society of Clinical Oncology, aims to establish a common framework for the appropriate clinical use of MRD testing in a tumor type-agnostic manner. It synthesizes currently available evidence, reviews region-specific clinical trends, addresses critical clinical questions related to MRD testing, and offers recommendations to guide healthcare professionals, biotechnology and pharmaceutical companies, and regulatory authorities. These recommendations were developed based on a voting process involving 15 expert members, ensuring a consensus-driven approach. These findings underscore the importance of collaborative efforts among various stakeholders in enhancing the clinical utility of MRD testing. This project aimed to foster consensus and provide clear guidelines to support the advancement of precision medicine in oncology and improve patient outcomes in the context of perioperative care.

## Introduction

In Japan, approximately 380,000 people die annually from malignant cancer, making it the leading cause of death. The 5-year relative survival rates for patients with solid tumors confined to primary sites and regions were 92.4% and 58.1%, respectively, whereas that for patients with distant metastases was 15.7% [1]. As resectable solid tumors confined to primary sites and regions are expected to be curable, operative treatment, in combination with chemotherapy and radiotherapy, is the recommended standard of care in these cases.

Recently, it has been reported that the detection of molecular residual disease (MRD) using circulating tumor DNA (ctDNA) analysis may be useful in assessing the postoperative recurrence risk of solid tumors. Some MRD tests are already in clinical use in Europe and the U.S., and the granting of regulatory approval and inclusion in insurance coverage is expected in Japan in the future. Nonetheless, although MRD testing is expected to improve the clinical management of resectable solid tumors, the following issues regarding its clinical implementation need to be addressed:

1. Awareness of MRD testing is low among both medical professionals and patients.
2. A variety of MRD testing assays and methods are not standardized.
3. The high cost of MRD testing has a significant impact on medical economics.
4. There is a lack of high-level evidence from large-scale studies, including clinical studies and randomized trials.
5. Consistent interpretation of the data is difficult because the standard of care can vary considerably depending on tumor type.
6. There are currently no guidelines for the appropriate clinical use of MRD testing.

Therefore, it is necessary to formulate a common view of MRD testing in a tumor type-agnostic manner and prepare detailed guidelines for decision-making regarding its appropriate clinical use by medical professionals, biotechnology and pharmaceutical companies, regulatory authorities, and patients.

The objective of this document is to discuss and interpret the current evidence on MRD testing comprehensively and to reach a common view of its appropriate clinical use in a tumor type-agnostic manner.

*Overview*

Chapter 2 provides an overview of ctDNA analysis and MRD testing.

Chapter 3, particularly region-specific clinical trends, provides a comprehensive review of the current evidence for each tumor type.

Chapter 4 presents Clinical Questions (CQs) related to the appropriate clinical use of MRD testing, as well as the corresponding current tumor type-agnostic recommendations. With regard to these recommendations, we prioritized consensus building to obtain common views while also considering that the evidence for standards of care and MRD testing vary depending on the tumor type. Therefore, it should be noted that the details of the recommendations must be interpreted with reference to the relevant guidelines and other requirements pertinent to the respective tumor types.

Finally, in Chapter 5, the reference data and information on the latest MRD assays are provided.

When this position paper was planned, MRD testing had not obtained regulatory approval. The Japanese insurance system did not cover it, and certain recommendations for CQs are based on insufficient evidence at this time. Therefore, the descriptions and recommendations provided here could change significantly with the accumulation of future evidence. Considering that regulatory approval for MRD testing was not taken into account in preparing these recommendations and that they are based on existing evidence and consensus among the Working Group members, this document is characterized as a "position paper on appropriate clinical use."

## Overview

### ctDNA

Liquid biopsy refers to the use of body fluid samples for analysis of tumor-derived biomolecules. Various body fluids, such as blood, saliva, urine, cerebrospinal fluid, bile, and pancreatic juice, are used as specimens for analysis. Of these, blood can be collected repeatedly in a minimally invasive manner. Moreover, blood sample collection methods are well standardized, and the blood contains biomolecules derived from many organs. Therefore, blood sample analysis has been clinically used for many tumor types [2, 3].

The presence of extracellular DNA, also called cell-free DNA (cfDNA), in blood was first reported in 1948 [4]. Although it is present only in trace amounts (1–10 ng in 1 mL of plasma) in healthy individuals, its amount is reported to increase under certain conditions, such as cancer and infection, and after cerebral infarction, traumatic injury, and excessive exercise. ctDNA is a form of cfDNA released into the blood as a result of the apoptosis or necrosis of cancer cells. The reported variant allele frequency (VAF) of ctDNA in cfDNA is approximately 0.1–10% [2]. Since it was first reported in 1994 that *KRAS* mutations identical to those in tumor tissues were detectable in the plasma cfDNA of a patient with pancreatic cancer, research on ctDNA has advanced rapidly [5].

As ctDNA is present in plasma only in trace amounts, standardization of preanalytical conditions is essential for appropriate analysis [6]. An important consideration in this regard is that if leukocytes are lysed due to sample blood coagulation, the resulting increase in normal cfDNA levels makes it difficult to detect ctDNA; therefore, blood collection tubes containing anticoagulants need to be used for such analyses. Ethylenediaminetetraacetic acid (EDTA) is the preferred anticoagulant, whereas heparin is not appropriate. Furthermore, to remove blood cells that may dilute cfDNA, it is important to ensure that the initial centrifugation of the blood collected in EDTA-containing collection tubes is performed within a few hours of sample collection. Specially designed collection tubes that minimize the incorporation of leukocyte-derived cfDNA are often used, and blood samples can be stored at room temperature for up to 14 days. For other precautions regarding ctDNA analysis according to the specific methodology used, please refer to the position paper of the Japanese Promotion Council for Laboratory Testing [6].

As ctDNA has a half-life of less than an hour, it essentially reflects the real-time whole-body tumor status in clinical practice [2, 3]. Therefore, whereas biopsies are more invasive and genomic analysis of tissue biopsies is associated with the problem of spatial and temporal genomic heterogeneity in tumor tissues, ctDNA has the advantage of allowing real-time, minimally invasive, and simpler genomic analysis of the entire tumor. In one study, a comparison of tumor tissue- and ctDNA-based genomic analyses of unresectable gastrointestinal cancer showed that ctDNA analysis had a significantly higher success rate (99.9% vs 89.4%, *P* < 0.001), required fewer days for analysis (7 days vs 19 days, *P* < 0.001), and had a higher enrollment rate in the genomic analysis clinical study (9.5% vs 4.1%, *P* < 0.001). Moreover, the response rates for both analyses were comparable (20.0% vs 16.7%, *P* = 0.69), further highlighting the utility of ctDNA for genomic analysis [7].

### MRD

MRD can represent “molecular residual disease,” “minimal residual disease,” or “measurable residual disease,” and a literature search revealed that MRD was used for “minimal residual disease” in 74%, “molecular residual disease” in 21%, and “measurable residual disease” in 5% of reports, highlighting the discrepancies in the definition of this term [8]. “Minimal residual disease” or “measurable residual disease” can be defined as a form of hematologic malignancy that is below the limit of detection of optical microscopy but is detectable using techniques such as multi-parameter flow cytometry and next-generation sequencers (NGS). Accordingly, it is considered an important hematologic prognostic factor for malignancy and is recommended to be evaluated over time during treatment [9]. In 2018, the US Food and Drug Administration (FDA) approved blinatumomab therapy for patients with adult acute lymphocytic leukemia and minimal residual disease after chemotherapy. Therefore, minimal residual disease has been established as a new condition requiring therapeutic intervention.

Conversely, “molecular residual disease” can be defined as evidence of recurrence at the molecular level that is detectable prior to the clinical, biological, or radiological evidence of recurrence. It is evaluated by detecting ctDNA in the postoperative recurrence-free state of solid tumors. The term “molecular residual disease” was first reported in 2008 in the context of detection after resection for colorectal cancer and its correlation with recurrence [10]. Since then, research on tumor type-agnostic MRD in solid tumors has advanced rapidly. This document provides expert opinions on the evaluation and appropriate clinical use of MRD—defined as “molecular residual disease”—by ctDNA analysis in solid tumors as a whole. MRD is the presence of detectable ctDNA in the absence of findings suggesting postoperative recurrence on imaging, and cases of ctDNA detected before and after surgery (referred to as preoperative ctDNA-positive and postoperative MRD-positive, respectively) are distinguished.

ctDNA analysis is used for both comprehensive genomic profiling (CGP) and MRD testing. CGP refers to the comprehensive analysis of a large number of genomic alterations using NGS or other methods (reported limit of detection [LOD] for VAF, 0.1–1%). On the other hand, MRD testing uses techniques such as molecular barcoding, BEAMing (beads, emulsions, amplification, and magnetics), and droplet digital polymerase chain reaction (ddPCR) to analyze a smaller number of specific genes or molecular markers (LOD, about 0.01–0.1%). It is characterized mainly by its precision [3]. Therefore, CGP is mainly used for decision-making regarding drug therapies for genomic alterations detected in patients with advanced, recurrent cancer, whereas MRD testing is mainly used to evaluate the risk of recurrence and diagnose recurrence at the molecular level in an apparently recurrence-free state after curative-intent treatment. Both CGP and MRD testing utilize ctDNA analysis; however, it is necessary to understand the aforementioned differences when considering their clinical applications.

MRD testing can be classified into two types: tumor-informed, which requires tumor tissue, and tumor-naïve, which does not require tumor tissue [3]. In tumor-naive assays, prespecified gene panels are used to detect MRD by analyzing epigenomic alterations in ctDNA without using any tumor tissue. For tumor-informed assays, two modes of testing are available: detection of MRD using a patient-specific panel designed based on the genomic analysis of tumor samples obtained by biopsy or surgery and detection of variants using tumor tissue and a standard gene panel. Tumor-informed assays that use a patient-specific panel are highly sensitive but require more time and depend on the quality and quantity of the tumor specimen. However, tumor-naive assays do not require tumor tissue, and the results can, therefore, be obtained faster.

As of August 2024, no clinical MRD assay has been approved in Japan. In the US, Signatera (Natera Inc., Austin, TX, USA) and RaDaR (NeoGenomics Inc., Fort Myers, FL, USA) tumor-informed assays using patient-specific panels and Guardant Reveal (Guardant Health Inc., Redwood City, CA, USA) tumor-naive assays are covered by Medicare and Medicaid reimbursement under the approval of the Centers for Medicare and Medicaid Services (CMS). Signatera and RaDaR are also certified with the Conformité Européenne (CE) marking in Europe and are available for clinical use there. For more information on the individual assays, please refer to "Chapter 5. Reference data."

### MRD testing: Japanese and international guidelines

The Precision Medicine Working Group of the European Society of Medical Oncology (ESMO) discussed the clinical use of MRD testing in its 2022 report on the use of ctDNA assays. It states that the specificity of MRD-positive recurrence is 90% and that the lead time from MRD detection to clinical recurrence may be as high as 6 months. Although the report acknowledged the clinical validity of MRD testing in terms of recurrence prediction, it also pointed out the importance of developing ultrasensitive MRD assays with LOD lower than 0.01% because the sensitivity is currently inadequate (at less than 50%) [11]. Furthermore, regarding the clinical utility of MRD testing, the report highlighted the importance of demonstrating prognostic improvement and safe treatment simplification by therapeutic interventions based on MRD testing results through randomized clinical trials. The report does not mention concrete CQs or recommendation levels regarding MRD testing because the available evidence was considered insufficient. Similarly, the 2024 US NCCN Guidelines Colorectal Cancer Panel did not recommend the routine use of MRD testing because the data on curative therapeutic interventions for MRD-positive patients were considered insufficient. Accordingly, enrollment in clinical studies on MRD testing is increasing [12]. ctDNA analysis was also covered by panels for other tumor types; however, there was no mention of MRD testing in August 2024.

On the other hand, the 2023 "Molecular Testing for Colorectal Cancer Treatment (5th Edition)" in Japan specified the following basic requirements: "Panel tests for the detection of the minimal residual tumor should be performed in colorectal cancer patients who have undergone curative-intent resection, for treatment selection according to the risk of recurrence" [13]. Notably, MRD testing is "strongly recommended" as a technique that can be performed repeatedly to identify high-risk groups for recurrence because its clinical utility has been demonstrated in prospective phase II clinical studies.

In summary, although there were differences in the recommendation levels for MRD testing among the Japanese, American, and European guidelines, there was agreement to a certain degree that while MRD testing has some clinical validity, further evidence is required to establish its clinical utility, such as in terms of improving prognosis. Currently, several randomized clinical studies are ongoing worldwide to evaluate the utility of therapeutic interventions based on MRD testing results, warranting the need for a position paper that reviews the updated results in a timely manner.

## Particulars: regions-specific clinical trends

### Objectives

This position paper serves as a guideline for the appropriate clinical use of tumor-type agnostic MRD testing. MRD testing was first reported for colorectal cancer in 2008 [10]. Since then, considerable evidence has accumulated on this topic, and it is expected to be more widely used clinically in the future. However, some tumor types lack adequate evidence, and more comprehensive clinical studies are required.

To reach a consensus regarding tumor type-agnostic MRD testing, it is necessary to fully understand and discuss the clinical trends corresponding to each region. In this chapter, experts in each field comprehensively reviewed the currently available evidence and ongoing clinical studies to promote the appropriate clinical development and use of MRD testing in each region.

### Gastrointestinal cancer

#### Status of evidence accumulation

A PubMed literature search using the keywords “colorectal cancer,” “gastric cancer,” “esophageal cancer,” and “circulating tumor DNA" OR “surgery” for each of them yielded 513, 131, and 86 reports published till June 2024, respectively. Although there were some limitations in terms of the search terms, most reports were related to colorectal cancer. Furthermore, 157, 54, and 18 reports were published before 2019; 224, 49, and 41 were published between 2020 and 2022; and 132, 28, and 27 were published in or after 2023, respectively, showing an increasing trend in the number of reports for these cancers year by year. This indicates that there is growing concern about and interest in MRD testing based on ctDNA analysis for gastrointestinal cancers.

#### Major literature reports (Table 1)

**Table 1:** Major literature reports in each region

Target tumor	Author	Year of publication	Journal	PMID	Sample size	Median observation period (months)	Analysis method	Assay method	Target stage (UICC)
Colorectal	Tie J et al.	2016	Sci Transl Med	27,384,348	230	27	NGS	Tumor-informed	II
Colorectal	Tie J et al.	2019	JAMA Oncol	31,621,801	96	28.9	NGS	Tumor-informed	III
Colorectal	Tarazona N et al.	2019	Ann Oncol	31,562,764	150	24.7	ddPCR	Tumor-informed	
Colorectal	Reinert T et al.	2020	JAMA Oncol	31,070,691	130	12.5		Signatera^™^	I–III
Colorectal	Parikh AR et al.	2021	Clin Cancer Res	33,926,918	103	632.5 (days)		Guardant Reveal^™^	I–IV
Colorectal	Chen G et al.	2021	J Hematol Oncol	34,001,194	240	27.4		GeneseeqPrime^™^	II/III
Colorectal	Benhaim L et al.	2021	Eur J Cancer	34,731,746	184	NA	ddPCR	Tumor-informed	II–III
Colorectal	Henriksen TV et al.	2022	Clin Cancer Res	34,625,408	168	35		Signatera^™^	III
Colorectal	Tie J et al.	2022	N Engl J Med	35,657,320	459	37	NGS	Tumor-informed	II
Colorectal	Mo S et al.	2023	JAMA Oncol	37,079,312	350	21		ColonAiQ™	I–III
Colorectal	Ryoo SB et al.	2023	Br J Cancer	37,280,413	98	36.3		AlphaLiquid^®^Detect	II–III
Colorectal	Kotani D et al.	2023	Nat Med	36,646,802	1039	16.7		Signatera^™^	I–IV
Colorectal	Wenhua Fan	2024	Ther Adv Med Oncol	38,282,662	309	19.5	NGS	Tumor-informed	I–IV
Colorectal	Henriksen TV et al.	2024	Ann Oncol	37,992,872	851	26	dPCR	Tumor-informed	II–III
Colorectal	Parikh AR et al.	2024	Clin Cancer Res	38,695,832	52	31		Guardant Reveal^™^	IV
Colorectal	Tie J et al.	2019	Gut	29,420,226	159	24	NGS	Tumor-informed	
Colorectal	Khakoo S et al.	2020	Clin Cancer Res	31,852,830	47	26.4	ddPCR	Tumor-informed	
Colorectal	Zhou J et al.	2021	Clin Cancer Res	33,046,514	106	18.8	NGS	Tumor-informed	
Colorectal	McDuff et al.	2021	JCO Precis Oncol	34,250,394	29	20	ddPCR	Tumor-informed	II/III
Gastric	Yuan SQ et al.	2023	Cancer Commun	37,837,629	100	52.2		GeneseeqPrime^™^	II/III
Gastric	Yang J et al.	2020	Cell Death Dis	32,393,783	46	29.1	NGS	Tumor-informed	I–III
Gastric	Leal A et al.	2020	Nat Commun	31,988,276	50	42	NGS	Tumor-naïve	IB–IVA
Esophageal	Huffman et al	2022	JCO Precis Oncol	36,480,779	295	8.3		Signatera^™^	I–III
Esophageal	Ococks E et al.	2021	Ann Oncol	33,359,547	97	32.9		AVENIO^®^ ctDNA Surveillance Kit	
Esophageal	Azad TD et al.	2020	Gastroenterology	31,711,920	45	NA	NGS	Tumor-informed	IA–IIIB
Esophageal	Ng HY	2023	JAMA Surg	37,728,901	74	NA		AVENIO^®^ ctDNA Surveillance Kit	
Esophageal	Yue P et al.	2024	Mol Cancer	38,730,415	38	17	NGS	Tumor-informed	
Esophageal	Chen B et al.	2024	Nat Commun	38,429,311	42	27.6		GeneseeqPrime^™^	I–IV
Lung	Oh Y et al.	2024	Thorac Cancer	38,558,374	36	NA		Signatera^™^	I–IV
Lung	Tan AC et al.	2024	Cancer	38,422,026	57	40.8		Signatera^™^	IA–IIIB
Lung	Tian X et al.	2024	Thorac Cancer	38,409,945	58	31.1	NGS	Tumor-informed	I–III
Lung	Tran HT et al.	2024	Ann Oncol	37,992,871	85	51.6	NGS	Tumor-naive	I–III
Lung	Martin TK et al.	2024	J Thorac Cardiovasc Surg	38,244,856	108	16.4		Signatera^™^	I–II
Lung	Liu SY et al.	2023	Signal Transduct Target Ther	38,057,314	52	25.1	NGS	Tumor-informed	IIA–IIIB
Lung	Chen K et al.	2023	Cancer Cell	37,683,638	181	35.2	NGS	Tumor-informed	I–III
Lung	Jung HA et al.	2023	J Thorac Oncol	37,308,037	278	62	ddPCR	Tumor-naive	IA–IIIA
Lung	Wang S et al.	2023	Cancer Res Commun	37,377,889	88	10.2–28.8		GeneseeqPrime^™^	I–III
Lung	Li Y et al.	2023	EBioMedicine	37,027,928	56	NA	NGS	Tumor-informed	
Lung	Fu R et al.	2023	Mol Oncol	36,732,646	184	16	NGS	Tumor-informed	0–III
Lung	Zhang X et al.	2023	Front Oncol	37,091,156	73	29	NGS	Tumor-informed	IA–IIIB
Lung	Abbosh C et al.	2023	Nature	37,055,640	197	55.2	NGS	Tumor-informed	I–III
Lung	Wang S et al.	2022	J Hematol Oncol	36,183,093	117	NA	NGS	Tumor-informed	I–III
Lung	Xia L et al.	2022	Clin Cancer Res	34,844,976	330	35.1	NGS	Tumor-informed	I–III
Lung	Zhang JT et al.	2022	Cancer Discov	35,543,554	261	19.7	NGS	Tumor-naive & informed	I–III
Lung	Vessies DCL et al.	2022	Mol Oncol	35,674,097	36	23		AVENIO^®^ ctDNA Surveillance Kit	IIA–IIIA
Lung	Gale D et al.	2022	Ann Oncol	35,306,155	88	17.9		RaDaR^™^	I–III
Lung	Li N et al.	2022	Cancer	35,076,939	119	30.7	NGS	Tumor-informed	IA–IIIA
Lung	Yue D et al.	2022	Transl Lung Cancer Res	35,280,315	22	17.7	NGS	Tumor-naive	I–III
Lung	Waldeck S et al.	2022	Mol Oncol	34,653,314	21	26.2	NGS	Tumor-informed	IA–IIIB
Lung	Qiu B et al.	2021	Nat Commun	34,799,585	91	NA	NGS	Tumor-informed	I–IV
Lung	Ohara S et al.	2020	Transl Lung Cancer Res	33,209,612	20	12	NGS	Tumor-informed	IIA–IIIA
Lung	Peng M et al.	2020	Front Oncol	33,042,842	77	46	NGS	Tumor-informed	I–IV
Lung	Yang W et al.	2020	Lung Cancer	32,623,075	82	22.8	NGS	Tumor-informed	MIA–IA
Lung	Chen K et al.	2019	Clin Cancer Res	31,439,586	26	17.5	NGS	Tumor-informed	I–III <
Lung	Chaudhuri AA et al.	2017	Cancer Discov	28,899,864	40	NA	NGS	Tumor-naive & informed	I–III
Lung	Deutsch JS et al.	2024	Nat Med	37,903,504	66	29.5		PCM^™^	I–III
Lung	Provencio M et al.	2022	J Clin Oncol	35,576,508	43	NA		Oncomine^™^ Pan-Cancer Cell-Free Assay	IIIA
Lung	Wang Y et al.	2024	Clin Transl Med	38,450,838	105	NA	NGS	Tumor-naïve	II–IIIC
Lung	Nielsen LR et al.	2024	Cancer Treat Res Commun	38,428,066	56	NA		AVENIO^®^ ctDNA Surveillance Kit	IIB–IV
Lung	Yang Y et al.	2024	Cancer Lett	38,101,608	70	NA		Radiotron^®^	II–III
Lung	Pan Y et al.	2023	Cancer Cell	37,816,331	139	NA	NGS	Tumor-informed	IIB–IIIC
Lung	Provencio M et al.	2021	Lung Cancer	33,453,470	24	NA		Oncomine^™^ Focus Assay	IIIA–B
Lung	Knapp B et al.	2022	Front Oncol	35,419,282	43	NA		InVisionFirst^®^-Lung	II–III
Lung	Moding EJ et al.	2020	Nat Cancer	34,505,064	65	NA	NGS	Tumor-naive & informed	IIB–IIIB
Breast	Maria C et al.	2024	Clin Cancer Res	38,078,899	22	32.5		RaDaR^™^	II–III
Breast	Jacqueline AS et al.	2024	JCO Precis Oncol	38,691,816	156	58		Signatera^™^	I–III
Breast	Marla LS et al.	2022	J Clin Oncol	35,658,506	103	145.6		RaDaR^™^	II–III
Breast	Milan R et al.	2020	JAMA Oncol	32,644,110	196	17.2	NGS	Tumor-informed	II–III
Breast	Heather AP et al.	2020	Clin Cancer Res	32,170,028	142	85.2	NGS	Tumor-informed	0–III
Breast	Raoul CC et al.	2019	Clin Cancer Res	30,992,300	49	NA		Signatera^™^	I–III
Breast	Isaac GM et al.	2019	JAMA Oncol	31,369,045	170	35.5	dPCR	Tumor-informed	II–III
Breast	Yu HC et al.	2017	NPJ Breast Cancer	28,685,160	38	24	NGS	Tumor-informed	II–III
Breast	Fiegl H et al.	2005	Cancer Res	15,734,995	148	43.2		MethyLight	I–III
Breast	Tae HL et al.	2024	Cancer Res Treat	37,946,409	11	48		LiquidSCAN v2-PanCancer panel GENINUS	III (NAC)
Breast	Magbanua et al.	2023	Cancer Cell	37,146,605	84	57.6		Signatera^™^	II–III (NAC)
Breast	Frederic C et al.	2022	JCO Precis Oncol	36,170,624	44	36.4		Signatera^™^	I–III (NAC)
Breast	Po HL et al.	2021	Front Oncol	34,868,925	95	61.2		QIAseq Targeted DNA Panel	II–III (NAC)
Breast	Ortolan E et al.	2021	ESMO Open	33,743,331	42	36		Oncomine^™^ Pan-Cancer Cell-Free Assay	II–III (NAC)
Breast	Li S et al.	2020	JCO Precis Oncol	32,923,909	52	46	NGS	Tumor-naive	I–III (NAC)
Breast	Magbanua MJM et al.	2020	Ann Oncol	33,232,761	295	37.2		Signatera^™^	II–III (NAC)
Breast	Cavallone L et al.	2020	Sci Rep	32,895,401	26	63	ddPCR	Tumor-informed	I–III (NAC)
Bladder	Nordentoft I et al.	2024	Eur Urol	38,811,314	112	53.6	NGS	Tumor-informed	
Bladder	Ben DR et al.	2024	Eur Urol Oncol	38,521,660	112	8		Signatera^™^	I–IV
Bladder	Chauhan PS et al.	2023	NPJ Precis Oncol	36,658,307	74	23	NGS	Tumor-naïve	
Bladder	Szabados B et al.	2022	Eur Urol	35,577,646	36	25		Signatera^™^	
Bladder	Powles T et al.	2021	Nature	34,135,506	581	21.9		Signatera^™^	
Bladder	Christensen E et al.	2019	J Clin Oncol	31,059,311	68	21		Signatera^™^	
Bladder	Birkenkamp D et al.	2018	Eur Urol	28,958,829	60	NA	ddPCR	Tumor-informed	I–IV
Bladder	Christensen E et al.	2017	Eur Urol	28,069,289	27	NA	ddPCR	Tumor-informed	
Renal pelvis/ureter	Tamura D et al.	2024	Cancer Sci	38,083,992	23	24.7	ddPCR	Tumor-informed	
Renal pelvis/ureter	Nakano K et al.	2022	Cancer Sci	35,293,110	17	NA		Oncomine^™^ Pan-Cancer Cell-Free Assay	0a–IV
Renal	Park JS et al.	2024	Cancer Sci	38,475,661	48	31.8	NGS	Tumor-informed	
Renal	Buttner T et al.	2024	Am J Transl Res	38,322,559	45	63	RT-PCR	Tumor-naive	I–IV
Renal	Jung M et al.	2019	Clin Chem	30,626,634	100	NA	RT-PCR	Tumor-naive	I–IV
Prostate	Pope B et al.	2024	Eur Urol	38,378,299	118	35	NGS	Tumor-informed	
Prostate	Fei X et al.	2023	Cancer Res Treat	36,915,250	131	10	NGS	Tumor-informed	
Prostate	Lau E et al.	2020	Genome Med	32,807,235	189	NA	PCR	Tumor-naive	
Liver	Shen T et al.	2020	Liver Int	32,594,568	275	33	ddPCR	Tumor-naive	I–IV
Liver	Li CL et al.	2020	Hepatology	32,171,027	50	NA	ddPCR	Tumor-naive	
Liver	Cai Z et al.	2019	Clin Cancer Res	31,217,202	34	NA	NGS	Tumor-informed	
Liver	Wang D et al.	2020	Liver Int	32,279,416	40	NA	NGS	Tumor-informed	
Liver	Fu Y et al.	2022	Hepatol Int	35,674,872	258	NA	NGS	Tumor-naive	
Liver	Huang A et al.	2024	Hepatol Int	37,980,313	74	17	NGS	Tumor-informed	
Liver	Ye K et al.	2022	Front Oncol	35,311,090	96	NA	NGS	Tumor-naive	
Liver	Zhao L et al.	2022	Clin Transl Med	35,384,341	66	NA	NGS	Tumor-naive	
Liver	Zhu GQ et al.	2022	Mol Oncol	34,543,520	41	17.7		AVENIO^®^ ctDNA Surveillance Kit	
Biliary tract	King G et al.	2023	Ann Oncol	-	12	NA		Signatera^™^	I–III
Pancreatic	Kitahata Y et al.	2022	Ann Surg Oncol	34,724,126	27	14.4	ddPCR	Tumor-naive	
Pancreatic	Yang X et al.	2018	Transl Cancer Res	-	35	12.4	dPCR	Tumor-naive	I–III
Pancreatic	Nakano Y et al.	2018	Br J Cancer	29,360,815	45	NA	qPCR	Tumor-naive	I–II
Pancreatic	Kim MK et al.	2018	Clin Chem	29,352,043	41	NA	ddPCR	Tumor-naive	
Pancreatic	Groot VP et al.	2019	Clin Cancer Res	31,142,500	59	16	ddPCR	Tumor-naive	I–II
Pancreatic	Pietrasz D et al.	2017	Clin Cancer Res	27,993,964	31	33.3	ddPCR	Tumor-naive	I–II
Pancreatic	Lee B et al.	2024	J Clin Oncol	–	102	36	PCR	Tumor-naive	I–II
Pancreatic	Guo S et al.	2020	Br J Cancer	31,969,677	157	NA	ddPCR	Tumor-naive	I–III
Pancreatic	Hussung S et al.	2021	BMC Cancer	33,430,810	25	22	ddPCR	Tumor-naive	
Pancreatic	Lee B et al.	2019	Ann Oncol	31,250,894	38	38.4	dPCR	Tumor-naive	
Pancreatic	Jiang J et al.	2020	Front Oncol	32,850,360	27	18.6	ddPCR	Tumor-naive	I–IV
Pancreatic	Eckhoff AM et al.	2024	Ann Surg Oncol	38,170,407	35	13		Signatera™	
Pancreatic	Watanabe F et al.	2019	PLOS ONE	31,891,652	39	16.2	ddPCR	Tumor-naive	I–II
Pancreatic	Watanabe K et al.	2022	Int J Mol Sci	36,232,820	145	NA		Oncomin^™^ Pan-Cancer Cell-Free Assay & Tumor-informed	0–IV
Pancreatic	Okada T et al.	2020	J Gastroenterol	32,939,577	66	NA	dPCR	Tumor-naive	
Pancreatic	Ako S et al.	2021	Cancer Biol Ther		33	26.2	ddPCR	Tumor-naive	I–III
Pancreatic	Hadano N et al.	2016	Br J Cancer	27,280,632	105	54 (mean)	ddPCR	Tumor-naive	I–II
Pancreatic	Sausen M et al.	2015	Nat Commun	26,154,128	51	32	ddPCR	Tumor-naive	II
Pancreatic	Hata T et al.	2023	J Hepatobiliary Pancreat Sci	36,408,698	66	NA	ddPCR	Tumor-naive	
Pancreatic	Yamaguchi T et al.	2021	Ann Surg Oncol	33,128,119	97	29	ddPCR	Tumor-naive	I–II
Pancreatic	Lee JS et al.	2022	Clin Chem	36,177,751	70	11.4		AVENIO^®^ ctDNA Surveillance Kit	I–III
Pancreatic	Botta GP et al.	2024	Oncologist	39,022,993	100	13		Signatera^™^	I–III
Ovarian	Hou JY et al.	2022	Gynecol Oncol	36,117,009	69	24		Signatera^™^	I–IV
Ovarian	Chao A et al.	2023	Biomed J	36,208,860	29	33.15		ACT Monitor^®^	I–IV
Ovarian	Zhu JW et al.	2023	Int J Mol Sci	37,762,691	48	NA		SureSelect XT assay	I–IIIC
Ovarian	Heo J et al.	2024	Cancer Res	38,038,965	201	12.7	NGS	Tumor-naïve	I–IV
Ovarian	Kallio HM et al.	2024	Life Sci Alliance	38,580,393	63	4.4 (years)		xGen Inherited Disease panel	I–IV
Endometrial	Ashley CW et al.	2023	Clin Cancer Res	36,007,103	44	33		MSK-ACCESS	I–IV
Endometrial	Recio F et al.	2024	Gynecol Oncol	38,262,240	101	5.7		Signatera^™^	I
Cervical	Jeannot E et al.	2021	Clin Cancer Res	34,210,686	94	16.7	ddPCR	Tumor-naive (HPV E7)	I–IV
Cervical	Han K et al.	2023	J Clin Oncol	37,972,346	60	2.2 (years)	ddPCR, NGS	Tumor-naive (HPV)	I–IV
Pharyngeal	Chan ATC et al.	2002	J Natl Cancer Inst	12,419,787	170	116 (weeks)	qPCR	Tumor-naive (EBV-DNA)	I–IV
Pharyngeal	Lin JC et al.	2004	N Engl J Med	15,190,138	99	30	qPCR	Tumor-naive (EBV-DNA)	III–IV
Pharyngeal	Li et al.	2017	Chin J Cancer	29,116,021	385	52.8	qPCR	Tumor-naive (EBV-DNA)	I–IVB
Pharyngeal	Chen FP et al.	2020	Cancer	32,125,701	1984	60	qPCR	Tumor-naive (EBV-DNA)	I–IVA
Pharyngeal	Chan DCT et al.	2022	Ann Oncol	35,491,007	769	5.1 (years)	qPCR, NGS	Tumor-naive (EBV-DNA)	IIB–IVB
Pharyngeal	Chera BS et al.	2020	J Clin Oncol	32,017,652	45	19.2	dPCR	Tumor-naive (HPV-DNA)	I–III
Pharyngeal	Rutkowski TW et al.	2020	J Transl Med	32,293,457	66	NA	qPCR	Tumor-naive (HPV-DNA)	
Pharyngeal	Tanaka H et al.	2021	Int J Cancer	32,895,945	35	21	ddPCR	Tumor-naive (HPV-DNA)	II–IV
Pharyngeal	Berger BM et al.	2022	Clin Cancer Res	35,576,437	1076	9		NavDx^®^	
Pharyngeal	Ferrandino RM et al.	2023	JAMA Otolaryngol Head Neck Surg	37,422,913	290	40.5		NavDx^®^	
Pharyngeal	Hanna GJ et al.	2023	Clin Cancer Res	37,566,241	543	27.9		NavDx^®^	
Head and neck	Flach S et al.	2022	Br J Cancer	35,132,238	17	371 (days)		RaDaR^®^	III–IVb
Head and neck	Honore N et al.	2023	Ann Oncol	37,879,442	53	31	NGS	Tumor-naive	I–IVB
Head and neck	Burgener JM et al.	2021	Clin Cancer Res	34,158,359	18	41.5	NGS	Tumor-naïve	
Head and neck	Kogo R et al.	2022	Cancer Med	35,352,507	24	563 (days)	dPCR	Tumor-informed	I–IV
Head and neck	Liu G et al.	2024	J Clin Oncol	-	130	NA	NGS	Tumor-naive	I–IVB
Skin	Lee RJ et al.	2018	Ann Oncol	29,112,704	161	60	ddPCR	Tumor-naive	II–III
Skin	Rowe SP et al.	2018	Mol Oncol	30,113,761	31	8.7	BEAMing	Tumor-naive	IIB–IV
Skin	Lee JH et al.	2019	Ann Oncol	30,860,590	119	26	ddPCR	Tumor-naive	IIIB/C/D
Skin	Tan L et al.	2019	Ann Oncol	30,838,379	133	18	ddPCR	Tumor-naive	III
Skin	Braune J et al.	2020	JCO Precis Oncol	35,050,727	8	NA	ddPCR	Tumor-naive	III
Skin	Forschner A et al.	2022	J Dtsch Dermatol Ges	35,555,861	5	NA	ddPCR	Tumor-naive	IIIC/D
Skin	Giunta EF et al.	2022	Cancers	35,804,825	26	26.7	qPCR	Tumor-naive	III–IV
Skin	Gouda MA et al.	2022	ESMO Open	34,942,440	23	NA	ddPCR	Tumor-naive	I–III
Skin	Brunsgaard EK et al.	2023	Melanoma Res	37,040,662	28	14.8		Signatera^™^	IIB–III
Skin	Eroglu Z et al.	2023	Cancer	36,869,646	30	19.6		Signatera^™^	III
Skin	Akaike T et al.	2024	J Clin Oncol	39,052,958	319	9.8		Signatera^™^	I–IV

Preoperative ctDNA/postoperative MRD positive rate			Median (units) or rate (%, duration) of recurrence/progression-free survival by preoperative ctDNA/postoperative MRD		Comparison of preoperative ctDNA/postoperative MRD positive vs. negative in terms of recurrence^a^				Other comments
Preoperative	Postoperative	Over time	Positive patients	Negative patients	HR or OR (95% CI)^b^	*P* value	Sensitivity	Specificity	
NA	8.7%	11%	0% (3 years)	90% (3 years)	18 (7.9–40)	< 0.001	48.0%	100%	
NA	21%	17%	47% (3 years)	76% (3 years)	3.8 (2.4–21.0)	< 0.001	42	NA	
63.8%	20.3%	34.0%	NA	NA	6.96 (2.57–18.91)	0.0001	82.4%	NA	
88.5%	10.6%	20%	NA	NA	7.2 (2.7–19.0)	< 0.001	87.5%	98.0%	
NA	24.0%	30.6%	168 (days)	NR	11.20	< 0.0001	55.6%	100.0%	
64.2%	8.3%	20%	39.3% (2 years)	89.4% (2 years)	10.98 (5.31–22.72)	< 0.001	82.6%	94.1%	
27.5%	10.5%	NA	NA	NA	3.22 (1.32–7.89)	0.00027	NA	NA	
91%	14%	19.3%	NA	NA	7.0 (3.7–13.5)	< 0.001	42.1%	95.1%	
NA	15.5%	NA	86.4% (3 years)	92.5% (3 years)	1.83 (0.79–4.27)	NA	NA	NA	
78.4%	23.1%	20.8%	NA	NA	17.5 (8.9–34.4)	< 0.001	78.0%	90.2%	
93.8%	21.4%	NA	32.2% (3 years)	88.0% (3 years)	8.40 (3.49–20.2)	< 0.001	61.9%	83.9%	
91.3%	18.0%	21.2%	38.4% (18 months)	90.5% (18 months)	10.0	< 0.0001	58.7%	91.5%	
NA	14.3%	17.5%	NA	NA	13.17 (5.54–31.29)	< 0.0001	64.6%	94.8%	
NA	7.2%	NA	NA	NA	11.3 (7.8–16.4)	< 0.001	35	98	
57.4%	45.2%	NA	6.6 (months)	27.3 (months)	5.27 (2.31–12.0)	< 0.0001	58.1	90.9	
77%	12%	NA	33% (3 years)	87% (3 years)	13.0 (5.5–31)	< 0.001	47.8%	94.1%	
74%	13%	NA	NA	NA	39.9 (4.0–399.5)	0.002	100%	100%	
75%	6.7%	NA	NA	NA	25.3 (1.475–434.0)	< 0.001	NA	NA	
34.6%	21.1%	NA	NA	NA	11.56	0.007	66.7%	100%	
38%	25%	NA	NA	NA	2.74 (1.37–5.48)	0.003	42.4%	83.6%	
45%	18%	38.6%	7.2 (months)	NR	6.56 (8.316–208.5)	< 0.0001	100.0%	84.0%	
54%	38%	NA	18.4 (months)	NR	3.0 (1.3–6.9)	0.01	NA	NA	
96%	23.5%	27.2%	6 (months)	NR	10.7 (4.3–29.3)	< 0.0001	62.5%	100%	
NA	20.3%	NA	8.7 (months)	26.7 (months)	5.35 (2.10–13.63)	≤ 0.0001	35%	97%	
60%	16.1%	NA	NA	NR	18.7 (1.1–316.5)	< 0.0001	71.4%	100%	Surgery, CRT
NA	18.4%	NA	NA	NA	4.47 (1.57–12.69)	0.006	NA	NA	
92%	33%	NA	NA	NA	NA	NA	NA	NA	
72.5%	27.0%	NA			2.879 (1.214–6.827)	0.012	87%	64%	CRT
NA	NA	25.0%	NA	NA	15.0 (1.0–253)	0.01	NA	NA	
26.3%	NA	NA	NA	NA	16.10 (1.63–158.9)	< 0.0001	NA	NA	
NA	31.0%	53.4%	NA	NA	4.6 (1.54–13.74)	0.006	NA	NA	
60.0%	NA	NA	NA	NA	2.8 (1.23–6.37)	0.01	NA	NA	
NA	5.8%	11.1%	NA	NA	53 (5.5–513)	< 0.0001	80%	95.6%	
89.5%	27.6%	NA	47.3% (18 months)	93.8% (18 months)	6.67 (1.06–25)	0.005	NA	NA	
41.7%	NA	NA	NA	NA	8.86 (3.72–21.1)	< 0.001	84.2%	NA	
NA	NA	NA	NA	NA	2.72 (1.29–5.73)	0.03	NA	NA	
NA	NA	NA	NA	NA	6.2 (2.3–16.8)	< 0.0001	31.6%	94.7%	
52.1%	19.6%	NA	NA	NA	7.6 (3.0–19.1)	< 0.0001	73.7%	97.3%	
NA	24.7%	NA	NA	NA	5.07 (2.33–11.01)	< 0.001	57.9%	87%	
NA	NA	NA	NA	NA	8.84 (3.41–22.9)	< 0.001	NA	NA	
NA	25.0%	NA	NA	NA	6.8 (3.7–12.3)	< 0.00001	49.0%	NA	
NA	NA	32.3%	NA	NA	3.9 (1.85–8.20)	0.00011	NA	NA	
20.9%	NA	NA	NA	NA	11.1 (6.5–19.0)	< 0.001	NA	NA	
36.4%	NA	NA	12 (months)	NR	0.08 (0.02–0.33)^b^	< 0.001	36.2%	NA	
NA	NA	NA	NA	NA	NA	0.24	NA	NA	
51.0%	NA	26.0%	NA	NA	14.8 (5.82–37.48)	< 0.00001	NA	100%	
24.8%	10.3%	31.1%	NA	NA	3.04 (1.22–7.58)	0.012	NA	NA	
86.0%	31.8%-37.5%	NA	8.5 (months)	NR	13.01 (1.49–113.23)	< 0.01	62.5%	85.7%	
57.0%	19.0%	NA	NA	NA	0.094 (0.01–0.061)^b^	0.013	NA	NA	
69.3%	21.2%	NA	NA	NA	3.95 (1.96–7.96)	< 0.001	NA	NA	
40.0%	20.0%	NA	NA	NA	NA	0.015	NA	NA	
59.7%	NA	NA	NA	NA	2.90 (1.333–6.319)	0.0035	NA	NA	
18.3%	NA	NA	NA	NA	8.5 (1.3–56.3)	0.02	NA	NA	
NA	NA	NA	NA	NA	7.55 (2.09–27.27)	0.002	NA	NA	
93.0%	53.0%	54.0%	NA	NA	43.4 (5.7–341)	< 0.001	NA	100%	
NA	NA	NA	NA	NA	0.66 (0.26–1.67)^b^	NA	NA	NA	
69.8%	NA	NA	NA	NA	0.26 (0.07–0.93)^b^	0.038	NA	NA	
NA	NA	NA	NA	NA	3.164 (1.767–5.667)	< 0.001	NA	NA	RT/CRT
67.0%	48.0%	41.0%	NA	NA	4.1 (1.7–10)	< 0.001	NA	NA	RT/CRT
71.4%	NA	NA	9.9 (months)	18 (months)	2.60 (1.20–5.66)	0.01	NA	NA	RT/CRT
72.9%	53.0%	NA	NA	NA	0.18 (0.12–0.28)^b^	< 0.001	NA	NA	RT/CRT
66.7%	46.7%	NA	8 (months)	18 (months)	0.05 (0.01–0.42)^b^	0.006	NA	NA	RT/CRT
65.1%	17.6%	NA	74 (months)	567 (months)	NA	0.01	NA	NA	RT/CRT
78.0%	50.0%	NA	NA	NA	NA	0.0006	100.0%	100.0%	RT/CRT
NA	47.9%	NA	NA	NA	NA	< 0.001	95.7%	91.0%	TNBC
NA	NA	NA	NA	NA	52.98 (18.32–153.20)	< 0.0001	88.2%	NA	All subtypes
NA	NA	10.0%	NA	NA	NA	NA	85.7%	97.4%	HR + HER2-
NA	NA	62–65%	32.5 (months)	NR	2.99 (1.38–6.48)	0.006	79.0%	NA	TNBC
NA	NA	NA	NA	NA	20.8 (7.3–58.9)	NA	81.0%	NA	All subtypes
NA	NA	33.0%	NA	NA	35.8 (7.9–161.3)	0.001	89.0%	100.0%	All subtypes
51.2%	NA	NA	NA	NA	16.7 (3.5–80.5)	0.001	96.0%	NA	All subtypes
NA	12.0%	NA	4.6 (months)	NR	12.6 (3.06–52.2)	0.0001	31.0%	100.0%	TNBC
NA	NA	22.3%	NA	NA	NA	0.015	NA	NA	HR + , HR-
NA	27.2%	NA	67% (4 years)	100% (4 years)	NA	0.032	NA	NA	TNBC
Baseline 73% After NAC 9%	NA	NA	NA	NA	10.4 (2.3–46.6)	NA	NA	NA	All subtypes
Baseline 58% After NAC 5%	NA	NA	NA	NA	53 (4.5–624)	0.01	NA	NA	All subtypes
Baseline 63% After NAC 33%	NA	NA	58% (5 years)	NA	4.29 (2.06–8.92)	0.0001	NA	NA	All subtypes
Baseline 47.8% After NAC 43%	NA	NA	40% (2 years)	83.9% (2 years)	2.65 (0.74–9.44)	NA	NA	NA	TNBC
Baseline 48%	NA	NA	NA	NA	5.72 (1.74–18.81)	0.011	NA	NA	All subtypes
Baseline HR + HER2- 69%, TNBC 91% After NAC HR + HER2- 12%, TNBC 22%	NA	NA	NA	NA	HR + HER2- 6.79 (3.10–14.84) TNBC 5.40 (2.72–10.72)	HR + HER2- < 0.0001 TNBC < 0.0001	NA	NA	HR + HER2-, TNBC
Baseline 96% After NAC 63%	NA	NA	NA	NA	3.45 (1.02–12.5)	0.046	NA	NA	TNBC
16.7%	25.9%	NA	NA	NA	23 (7.9–67.1)	< 0.0001	91.0%	92.0%	
50.5%	19.8%	NA	16.0% (1 year)	47.0% (1 year)	9.9 (2.6–37.0)	< 0.001	NA	NA	
NA	Urine 72.2%	NA	NA	NA	3	0.01	NA	NA	
Baseline 62.5% After NAC 46.7%	14.0%	NA	NA	NA	78.22 (8.64–707.78)	0.0001	NA	NA	
NA	37.0%	NA	NA	NA	6.3 (4.45–8.92)	< 0.0001	NA	NA	
NA	26.6%	NA	59% (1 year)	NA	NA	< 0.001	100.0%	98.0%	
NA	75.0%	NA	NA	NA	NA	0.001	NA	NA	
100.0%	NA	NA	NA	NA	NA	Plasma < 0.0001 Urine 0.031	NA	NA	
NA	Plasma 52.2% Urine 47.8%	NA	NA	NA	NA	NA	NA	NA	
NA	58.8%	NA	NA	NA	6.259 (1.485–26.38)	0.0125	NA	NA	
20.8%	NA	NA	NA	NA	NA	NA	NA	NA	
NA	37.8%	NA	NA	NA	NA	0.005	NA	NA	
12.0%	NA	NA	NA	NA	NA	NA	NA	NA	
16.0%	NA	NA	NA	NA	2.8 (1.1–7.1)	0.0055	NA	NA	
65.5%	NA	NA	NA	NA	0.14 (0.09–0.24)^b^	< 0.01	NA	NA	
12.0%	NA	NA	NA	NA	2.4 (1.2–4.8)	0.014	NA	NA	
20.4%	NA	NA	NA	NA	2.15 (1.58–2.93)	< 0.001	NA	NA	
97.7%	22.7%	NA	38.2% (1 year)	90.0% (1 year)	NA	< 0.0001	NA	NA	
100.0%	32.4%	50.0%	NA	NA	NA	< 0.0001	58.8%	NA	
97.5%	NA	NA	NA	NA	4.10 (1.58–10.67)	0.004	NA	NA	
96.5%	NA	NA	31.0% (1 year)	88.2% (1 year)	7.1 (3.2–16.0)	< 0.001	NA	NA	
47.0%	17.6%	NA	17.2 (month)	19.2 (month)	NA	< 0.001	NA	NA	
–	24.0%	NA	4 (months)	NR	4.551 (2.220–9.330)	< 0.0001	NA	NA	
–	35.6%	NA	NA	NA	11.77 (4.96–27.96)	< 0.0001	70.4%	93.8%	
63.4%	46.3%	NA	NA	NA	4.3	0.032	NA	NA	
100.0%	33.3%	NA	NA	NA	7.4 (2.6–4758)	0.00044	NA	NA	
59.3%	51.9%	NA	11.2 (months)	12.3 (months)	NA	0.3671	NA	NA	BR
65.7%	22.9%	NA	NA	NA	NA	0.001	NA	NA	
24.4%	44.4%	NA	NA	NA	NA	0.014	NA	NA	
36.6%	NA	NA	NA	NA	NA	0.016	NA	NA	
49.0%	26.8%	58.7%	5 (months)	15 (months)	NA	< 0.001	NA	NA	
NA	19.4%	NA	4.6 (months)	17.6 (months)	NA	0.03	NA	NA	
NA	39.2%	NA	13 (months)	22 (months)	0.28	0.003	NA	NA	
NA	11.5%	NA	12.1 (months)	25.4 (months)	7.294 (2.397–22.194)	< 0.001	NA	NA	
NA	48.0%	NA	NA	NA	1.18 (0.46–3.01)	0.7246	NA	NA	
62.2%	37.1%	NA	5.4 (months)	17.1 (months)	5.4 (1.9–15.2)	< 0.0001	57.0%	100.0%	
66.7%	29.6%	NA	NA	NA	5.2	0.019	NA	NA	
31.4%	15.1%	25.7%	3.2 (months)	9.8 (months)	8.1 (2.6–25.5)	< 0.001	NA	NA	
17.9%	7.7%	35.9%	NA	NA	NA	NA	NA	NA	
39.4% (Tumor-naive) 56.3% (Tumor-informed)	NA	NA	13.3 (months)	NR	NA	0.001	NA	NA	
NA	19.3%	NA	NA	NA	NA	NA	NA	NA	
NA	36.4%	NA	7.7 (months)	26.2 (months)	3.37 (1.36–8.35)	< 0.01	NA	NA	
31.4%	–	NA	6.1 (months)	16.1 (months)	NA	< 0.001	NA	NA	
56.9%	50.0%	NA	9.9 (months)	NR	NA	0.0199	NA	NA	
28.8%	24.2%	NA	NA	NA	2.110 (1.039–4.284)	0.034	NA	NA	
24.7%	27.8%	NA	6.9 (months)	19.2 (months)	NA	0.027	NA	NA	
37.7%	15.1%	19.5%	NA	NA	NA	0.06	NA	NA	
NA	29%	29.6%	6.4 (months)	33.3 (months)	5.45 (2.94–10.1)	< 0.0001	NA	NA	
73.0%	33.0%	23.0%	NA	NA	17.6 (3.2–97.4)	< 0.001	100.0%	100.0%	
58.6%	37.9%	9.1%	27.3% (2 years)	77.8% (2 years)	6.56 (1.07–40.17)	0.042	NA	NA	
NA	77.4%	NA	27.6% (5 years)	53.6% (5 years)	2.32 (0.67–8.05)	0.18	NA	NA	
69.2%	40.1%	21.8%	6.7 (months)	17.9 (months)	10.7	< 0.001	NA	NA	
93.0%	33.3%	NA	1.09 (years)	5.64 (years)	4.69	< 0.001	100.0%	NA	
22.0%	NA	24.0%	NA	NA	15.56 (2.16–112.16)	0.014	NA	NA	
NA	15.0%	24.0%	NA	NA	6.2 (1.5–26)	0.0006	NA	NA	Including sarcoma
63.0%	NA	NA	NA	NA	10.95 (2.94–40.7)	< 0.0001	NA	NA	Surgery, CRT
93.3%	NA	NA	15% (2 years)	82% (2 years)	8.58 (3.56–20.71)	< 0.001	NA	NA	CRT
NA	NA	NA	48% (1 year)	93% (1 year)	11.9 (5.53–25.43)	< 0.001	NA	NA	Nasopharyngeal, RT/CRT
NA	NA	NA	28.6% (2 years)	84.2% (2 years)	34.5 (7.4–162.1)	< 0.001	NA	NA	Nasopharyngeal, RT
69.4%	NA	24.2%	NA	NA	NA	NA	73.6%	87.2%	Nasopharyngeal, RT/CRT
NA	NA	38.7%	NA	NA	NA	NA	82.3%	80.0%	Nasopharyngeal, RT
NA	NA	NA	NA	NA	NA	NA	97.1%	NA	Nasopharyngeal, RT/CRT
NA	NA	18.0%	50% (2 years)	100% (2 years)	NA	< 0.0001	100.0%	100.0%	HPV + , oropharyngeal, SCC, CRT
NA	9.0%	–	NA	NA	NA	NA	100.0%	98.0%	HPV + , oropharyngeal, SCC, RT/CRT
NA	17.0%	–	33% (1 year)	93% (1 year)	NA	< 0.0001	NA	NA	HPV + , oropharyngeal, SCC, RT/CRT
NA	NA	7.4%	NA	NA	NA	NA	NA	NA	HPV + , oropharyngeal, SCC
NA	NA	NA	NA	NA	NA	NA	81.8%	100.0%	HPV + , oropharyngeal, SCC, surgery, RT/CRT
86.0%	NA	NA	NA	NA	22.5 (9.2–55.3)	< 0.0001	87.3%	99.4%	HPV + , oropharyngeal, SCC, surgery, CRT
100.0%	NA	NA	NA	NA	NA	NA	NA	NA	HPV-, Oral/orohypopharyngeal/ laryngeal, SCC
77.0%	41.4%	NA	23.5% (2 years)	86.6% (2 years)	4.34 (1.817–10.39)	0.011	77.8%	86.9%	Oral/orohypopharyngeal/ laryngeal, SCC, surgery, RT/CRT
NA	27.8%	NA	NA	NA	8.73 (1.5–50.92)	0.0046	NA	NA	HPV-, Oral/orohypopharyngeal/ laryngeal, SCC
NA	NA	NA	NA	NA	NA	< 0.0001	NA	NA	Oral/orohypopharyngeal/ laryngeal, SCC
NA	NA	NA	NA	NA	12.95 (4.51–37.22)	< 0.001	NA	NA	
NA	12.0%	NA	21% (5 years)	49% (5 years)	3.12 (1.79–5.47)	< 0.0001	18%	95%	Melanoma (*BRAF/NRAS*)
NA	3.4%	6.8%	NA	NA	NA	NA	NA	NA	Melanoma (*BRAF/NRAS*)
34%	NA	NA	17.6 (months)	49.4 (months)	2.11 (1.20–3.71)	< 0.01	NA	NA	Melanoma (*BRAF/NRAS/KIT*)
35%	24%	27%	3.5 (months)	13.5 (months)	10 (4.3–24)	< 0.001	55%	94%	Melanoma (*BRAF/NRAS/TERT/TP53/KIT*)
NA	62.5%	NA	NA	NA	NA	NA	83%	100%	Melanoma (*BRAF/NRAS*)
NA	20%	40.0%	NA	NA	NA	NA	20%	NA	Melanoma (*BRAF/NRAS/TP53*)
3.8%	NA	NA	NA	NA	NA	NA	11.1%	100%	Melanoma (*BRAF/NRAS*)
43.4%	31.8%	27.3%	26 (months)	NR (months)	NA	NA	80.0%	82.3%	Melanoma (*BRAF*)
46.4%	3.6%	21.4%	NA	NA	NA	NA	42.90%	100%	Melanoma
NA	17%	23%	4 (months)	NR	10.77 (1.77–65.57)	0.01	83%	96%	Melanoma
95.0%	10.0%	14.7%	26% (1 year)	79% (1 year	7.4 (2.7–20.3)	< 0.001	95.0%	90.0%	Merkel cell carcinoma

*PMID* PubMed Identifier, *UICC* Union for International Cancer Control, *ctDNA* circulating tumor DNA, *MRD* molecular residual disease, *HR* hazard ratio, *OR* odds ratio, *NGS* next-generation sequencing, *ddPCR* droplet digital polymerase chain reaction, *NA* not available, *NR* not reached, *dPCR* digital PCR, *qPCR* quantitative PCR, *NAC* neoadjuvant chemotherapy, *TNBC* triple negative breast cancer, *HR* hormone receptor, *HER2* human epidermal receptor 2, *BR* borderline resectable, *RT* radiation therapy, *CRT* chemoradiation therapy, *RT-PCR* reverse transcription PCR, *HPV* human papilloma virus, *EBV* Epstein-Barr virus, *SCC* squamous cell carcinoma, *BEAMing* beads, emulsions, amplification, and magnetics

^a^If analyzed at multiple time points, enter the data obtained postoperatively

^b^Values for MRD negative patients relative to those for MRD positive patients

##### Colorectal cancer

Colorectal cancer is the most studied gastrointestinal cancer in terms of MRD evaluation using ctDNA. One of the reasons for this is that colorectal cancer is an easily detectable cancer with high levels of ctDNA shedding into the blood. As described in the commentary on CQ1, the clinical validity of MRD testing is evaluated based on parameters such as sensitivity and specificity. Many studies have indicated that MRD positivity after curative-intent treatment predicts future clinical recurrence with high sensitivity and specificity, thereby demonstrating the clinical validity of MRD testing for this cancer type. Tie et al. evaluated MRD testing using the tumor-informed Safe-SeqS method in a prospective observational study that included 230 stage II patients and 96 stage III patients. They reported that postoperative and post-adjuvant treatment MRD positivity was a significant risk factor for recurrence [14, 15]. Parikh et al. used the Guardant Reveal assay in a prospective observational study and reported that the sensitivity and specificity for recurrence in postoperative stage I–IV patients were 55.6% and 100%, respectively (*N* = 103) [16]. Mo et al. reported a high risk of recurrence among postoperative MRD-positive patients using the ColonAiQ assay to detect cfDNA methylation in a prospective observational study (*N* = 299 stage I–III patients) [17]. Faulkner et al. conducted a meta-analysis of 37 studies (*N* = 3002 patients). They reported that postoperative MRD positivity was associated with an increased risk of recurrence regardless of cancer stage, including the presence or absence of adjuvant chemotherapy and assay type, and that it could serve as an independent indicator of poor prognosis (hazard ratio [HR]: 6.92, 95% confidence interval [CI] 4.49–10.64]) [18]. Studies in other countries have shown that MRD testing using ctDNA analysis may be useful. In the randomized phase II DYNAMIC study, 455 patients with pathological stage II were randomized into two groups: the standard group, wherein conventional clinicopathological factors were used as the eligibility criteria for adjuvant chemotherapy, and the MRD-guided group (based on MRD test results at 4 and 7 weeks after surgery). The results showed that the MRD-guided group was non-inferior to the standard group in terms of recurrence-free survival and had significantly reduced adjuvant chemotherapy use [19]. In the GALAXY study, a prospective observational study that included clinical stage II–IV patients, postoperative MRD positivity was significantly associated with an increased risk of recurrence in 2240 patients included in the interim analysis (HR: 11.99 [95% CI 10.02–14.35]). It was also suggested that adjuvant chemotherapy for postoperative MRD-positive patients may reduce the risk of recurrence (adjusted HR: 0.23 [95% CI 0.15–0.35]) [20]. Therefore, the utility of MRD for predicting recurrence has been reported in many studies, and several large-scale prospective studies on MRD are ongoing.

However, not all studies have indicated the utility of MRD. In the randomized phase II/III NRG-GI005 (COBRA) study, which used the Guardant Reveal for MRD testing, patients with stage II colon cancer and a low risk of recurrence were assigned to two groups: the MRD-guided group, in which adjuvant chemotherapy was administered to postoperative MRD-positive patients, and the standard group, in which follow-up was conducted based on clinicopathological factors. Of the 635 enrolled patients, 16 tested positive for MRD postoperatively, and resolution of MRD at 6 months was observed in three of seven patients (43%) in the untreated follow-up group. In contrast, in the group receiving adjuvant chemotherapy, resolution of MRD at 6 months was observed in 1 of 9 patients (11%). Therefore, owing to the failure to achieve the primary objective for phase II, the study was discontinued [21]. The results of this study indicate that MRD testing may not be demonstrably useful, depending on the accuracy of the assay and the specificity of the patient population. Therefore, all study results should be interpreted with caution.

##### Gastric cancer

Yang et al. reported that MRD positivity after curative-intent surgery, as assessed using a tumor-informed assay, was associated with recurrence and shortened recurrence-free survival (HR: 14.78 [95% CI 7.991–61.29]) in stage I–III patients (*N* = 46 patients) [22]. In the CRITICS study, Leal et al. analyzed ctDNA using a tumor-naive assay (VariantDx). They reported that preoperative ctDNA negativity was significantly more frequent among responders (*P* = 0.03). Recurrence-free survival was significantly shortened in postoperative MRD-positive patients (HR: 21.8 [95% CI 3.9–123.1]) [23]. Huffman et al. analyzed postoperative MRD positivity using the Signatera test. They reported that it was associated with a risk of recurrence and significantly shortened recurrence-free survival (HR: 23.6 [95% CI 10.2–66.0]) (*N* = 125 patients) [24]. Mi et al. conducted a meta-analysis of eight studies on gastric cancer (*N* = 423 patients). They reported that patients who were positive for ctDNA/MRD perioperatively were at a higher risk of recurrence. Those who were positive for preoperative ctDNA and postoperative MRD had significantly worse recurrence-free survival (HR: 6.37 [95% CI 2.70–15.01]) and overall survival (HR: 4.58 [95% CI 1.68–12.49]) [23]. Therefore, MRD has been reported to be useful for predicting the recurrence of gastric cancer. Prospective clinical studies, including MRD testing, have been conducted, and the accumulation of evidence and advancements in precision medicine applications are anticipated in the future.

##### Esophageal cancer

Owing to the nature of esophageal cancer treatment, there are very few reports on simple perioperative ctDNA/MRD evaluation, and most reports involve evaluations under neoadjuvant chemotherapy/chemoradiation therapy (CRT), adjuvant therapy, and curative-intent CRT. Azad et al. reported that ctDNA positivity after CRT significantly increased the risk of progressive disease and death (HR: 18.7 [95% CI 1.1–316.5]) and predicted recurrence earlier with higher sensitivity (71.4%) and specificity (100%) than clinical recurrence on imaging in squamous cell esophageal cancer (*N* = 45 patients) [25]. Ng et al. reported that MRD positivity at 6 months postoperatively was associated with overall survival (HR: 7.84 [95% CI 1.87–32.97]) in patients who underwent curative-intent resection (*N* = 74) [26]. Zhang et al. conducted a meta-analysis of 22 studies (*N* = 1144 patients) and reported that postoperative MRD positivity was significantly associated with overall survival (HR: 3.87 [95% CI 2.86–5.23]) and progression-free survival (HR: 4.28 [95% CI 3.34–5.48]) [27]. The utility of preoperative ctDNA and postoperative MRD testing has also been sporadically reported for esophageal cancer. Due to the epidemiological landscape of incidence, clinical studies are ongoing, mainly in Asian countries such as Japan and China. In Japan, neoadjuvant chemotherapy has been adopted as the standard of care. However, the impact of neoadjuvant chemotherapy on the interpretation of postoperative MRD results has not been clarified, and further studies are required.

#### Expected future clinical development (Table 2)

**Table 2:** Currently ongoing clinical studies in each region

Target tumor	Study title	Country	Study design	Sample size	Assay method	Major eligibility criteria	Study outline
Colorectal	VEGA (jRCT1031200006)	Japan	Interventional, phase III	1000	Signatera^™^	Stage II–III, postoperative, MRD negative	RCT of CAPOX vs. follow-up
Colorectal	ALTAIR (NCT04457297)	Japan	Interventional, phase III	240	Signatera^™^	Stage II–IV, post-curative treatment, MRD positive	RCT of FTD/TPI vs. placebo
Colorectal	SAGITTARIUS (NCT06490536)	Italy	Interventional, phase III	700	Signatera^™^	Stage II–III, postoperative	MRD( +): RCT of CAPOX 6 months vs. MRD-guided therapy MRD(-): RCT of standard of care vs. follow-up
Colorectal	NCT03803553	U.S	Interventional, phase III	400	Guardant Reveal^™^	Stage III, post-curative treatment	MRD( +): RCT of FOLFIRI vs. follow-up MRD(-): Follow-up MRD( +) with MSI-H, BRAF positive, or HER2( +): Targeted medication-assisted treatment
Colorectal	MIRROR (NCT06204484)	China	Interventional, phase III	349	NA (Burning Rock Dx)	Stage II–III, postoperative	MRD(-): RCT of CAPOX vs. MRD-guided therapy MRD( +): RCT of CAPOX vs. mFOLFOXIRI
Colorectal	AFFORD (NCT05427669)	China	Interventional, phase III	340	NA	Stage II–III, MRD positive	RCT of mFOLFOXIRI vs. mFOLFOX6
Colorectal	CLAUDIA (NCT05534087)	Korea	Interventional, phase III	236	NA	Stage II–III, on adjuvant chemotherapy, MRD positive	RCT of mFOLFOIRINIX vs. FOLFOX/CAPOX
Colorectal	CIRCULATE-US (NCT05174169)	U.S	Interventional, phase II/III	1912	Signatera^™^	Stage II–III, postoperative	MRD(-): RCT of CAPOX/mFOLFOX6 vs. follow-up MRD( +): RCT of CAPOX/mFOLFOX6 vs. mFOLFIRINOX
Colorectal	NRG-GI005(COBRA) (NCT04068103)	U.S	Interventional, phase II/III	635 (1408)	Guardant LUNAR1^™^	Stage IIA, postoperative	RCT of follow-up vs. MRD-guided therapy
Colorectal	COSMOS-CRC-03 (jRCT2072220055)	Japan	Interventional, phase II	160	Guardant Reveal^™^	Oligometastases, scheduled resection	Evaluation of the efficacy of MRD-guided therapy
Colorectal	AURORA (jRCT1071220087)	Japan	Randomized, phase II	90	Guardant Reveal^™^	Oligometastases, postoperative, MRD positive	RCT of mFOLFOX6 vs. mFOLFOXIRI + BEV
Colorectal	PEGASUS (NCT04259944)	Italy	Interventional, phase II	140	Guardant LUNAR1^™^	MSS, Stage II–III	Evaluation of the efficacy of MRD-guided therapy
Colorectal	ctDNA-nedCRC-lung (NCT05495672)	China	Interventional, phase II	100	OriMIRACLE S^™^	Suspected lung metastasis	Evaluation of the efficacy of MRD-guided therapy
Colorectal	IMPROVE-IT (NCT03748680)	Denmark	Interventional, phase II	64	NA	Stage I–II, postoperative, MRD positive	RCT of CAPOX vs. follow-up
Colorectal	NCT05699746	Shanghai, China	Interventional, phase II	38	MinerVa MRD assay	Stage I–II, postoperative, MRD positive	RCT of CAPOX vs. follow-up
Colorectal	NCT06358430	U.S	Interventional, phase I	42	NA	Postoperative, MRD positive	Evaluation of the efficacy of TROP2-CAR-NK therapy
Colorectal	NCT04920032	U.S	Interventional, phase I	22	Signatera^™^	Stage II–IV, post-adjuvant chemotherapy, MRD positive	Evaluation of the efficacy of FTD/TPI plus irinotecan
Colorectal	NCT05161585	China	Interventional	316	NA	Stage III	RCT of MRD surveillance vs. standard follow-up
Colorectal	IMPROVE-IT2 (NCT04084249)	Denmark	Interventional	250	NA	Stage II–III	RCT of MRD surveillance vs. standard follow-up
Colorectal	SYNCOPE (NCT04842006)	Finland	Interventional	93	NA	Rectal cancer, scheduled resection	RCT of TNT + MRD-guided therapy vs. long CRT
Colorectal	DAILY (NCT05036109)	U.S	Interventional	19	Signatera^™^	Postoperative, MRD positive	A lifestyle Intervention study
Colorectal	GALAXY (UMIN000039205)	Japan	Observational	6300	Signatera^™^	Stage II–III, scheduled resection	MRD monitoring
Colorectal	IMPROVE (NCT03637686)	Denmark	Observational	3182	NA	Stage I–III	MRD monitoring
Colorectal	BESPOKE CRC (NCT04264702)	U.S	Observational	1788	Signatera^™^	Stage I–IV	MRD monitoring
Colorectal	DANISH.MRD (NCT06076811)	Denmark	Observational	1600	NA	Stage I–III	MRD monitoring
Colorectal	Protector-C (NCT05444491)	China	Observational	1200	ColonAiQ^™^	Stage I–IV, scheduled resection	MRD monitoring
Colorectal	TRACC (NCT04050345)	U.K	Observational, interventional	1000	NA	Stage II–III, postoperative	Part B: Observational study Part C: RCT of standard of care vs. MRD-guided therapy
Colorectal	MiRDA-C Study (NCT04739072)	U.S	Observational	1000	NA	Stage II–IV, scheduled resection	MRD monitoring (+ analysis of RNA/proteomic analysis)
Colorectal	CORRECT-MRD II (NCT05210283)	U.S	Observational	750	Tumor-informed	Stage II–III, postoperative	MRD monitoring
Colorectal	GUIDE.MRD-01-CRC (NCT06111105)	Austria	Observational	590	NA	Stage III–IV	MRD monitoring
Colorectal	COSMOS-CRC-01 (UMIN000037765)	Japan	Observational	500	Guardant LUNAR^™^	Stage 0–II	MRD monitoring
Colorectal	CORRECT-MRD I (NCT06398743)	EU	Observational	400	Tumor-informed	Stage II–III, postoperative	MRD monitoring
Colorectal	CITCCA (NCT04726800)	Norway	Observational	300	NA (Clinical Genomics)	Stage I–III	MRD monitoring
Colorectal	pilotCRC-MRD (NCT06143644)	China	Observational	220	NA (Burning Rock Dx)	Stage I–IV, postoperative	MRD monitoring
Colorectal	FRENCH.MRD.CRC (NCT06287814)	France	Observational	70	NA	Stage III	MRD monitoring
Colorectal	FRENCH.MRD.CRLM (NCT06287723)	France	Observational	30	NA	Hepatic metastasis, scheduled resection	MRD monitoring
Gastric/ esophageal	CURE (NCT04576858)	Denmark	Observational	1950	NA	Post-resection/CRT	MRD monitoring
Gastric	TRINITY (NCT06253650)	Italy	Randomized, phase II	46	NA	HER2 + , postoperative, MRD positive	RCT of trastuzumab deruxtecan + capecitabine vs. FLOT
Gastric	MRD-GATE (NCT06157216)	China	Interventional, phase II	85	NA	Stage II–III	Utility evaluation of the MRD-guided therapy
Gastric	EXPLORING (NCT05494060)	China	Randomized, phase II	80	NA	Postoperative, MRD positive	RCT of penpulimab + anlotinib + XELOX vs. XELOX
Gastric	NCT03957564	China	Interventional, phase II	40	NA	Locally advanced, scheduled NAC	Utility evaluation of ctDNA, cfDNA, and CTC monitoring in NAC
Gastric	DECIPHER (NCT05965479)	U.K	Interventional, phase II	25	Signatera^™^	HER2 + , postoperative, MRD positive	Efficacy evaluation of of trastuzumab deruxtecan
Gastric	NCT06232395	China	Observational	1197	NA (Suzhou Huhu Health & Tec)	Postoperative	MRD monitoring
Gastric	NCT04511559	China	Observational	540	NA	Postresection	MRD monitoring
Gastric	COSMOS-GC-01 (UMIN000040148)	Japan	Observational	500	Guardant LUNAR^™^	Stage I–III (including GIST), scheduled resection	MRD monitoring
Gastric	NCT05029869	Vietnam	Observational	100	NA	Scheduled resection	MRD monitoring
Gastric	ZSGC-005 (NCT04000425)	China	Observational	55	AVENIO ctDNA surveillance kit	Scheduled resection	MRD monitoring
Esophageal	TRACT DNA (NCT05067842)	U.S	Observational	30	Signatera^™^	TNT + schedule resection	MRD monitoring
Esophageal	BEIR 2 (NCT06498752)	China	Interventional, phase II	102	NA	Stage I–IVa, post-CRT	Utility evaluation of the MRD-guided therapy
Esophageal	NCT05704530	Belgium	Interventional	248	NA	Locally advanced	MRD monitoring
Esophageal	DISCOVER study (jRCT1071240001)	Japan	Observational	200	Guardant Reveal^™^	Stage I–IV	MRD monitoring
Esophageal	NCT05759325	China	Observational	100	NA	Stage II–IVa	MRD monitoring
Esophageal	NATEC (NCT06103890)	China	Observational	100	NA	Stage II–III, scheduled NAC	MRD monitoring
Esophageal	ESO-Shanghai16 (NCT05426850)	China	Observational	100	NA	SCC, Stage II–IVB, scheduled CRT	MRD monitoring
Esophageal	ECMRD-001 (NCT05952661)	China	Observational	56	NA	Scheduled resection	MRD monitoring
Esophageal	RICE-MRD (NCT05900583)	China	Observational	50	NA (Haplox)	Stage II–IVa	MRD monitoring
Lung	ctDNA-Lung-Detect (NCT05254782)	Canada	Observational	360	RaDaR^™^	T1–T4N0, scheduled resection	MRD monitoring
Lung	ADAPT-E LUN0115 (NCT04585477)	U.S	Interventional, phase II	80	AVENIO ctDNA Surveillance Kit	Stage I–III, NSCLC, scheduled resection	MRD monitoring
Lung	ECTOP-1022 (NCT06323148)	China	Interventional, phase III	226	NA	Stage II–IIIA, EGFR + , NSCLC, scheduled resection	MRD( +): RCT of osimertinib vs. follow-up
Lung	NCT05536505	China	Interventional, phase II	180	NA	Stage IB–IIIB, EGFR + , NSCLC, postoperative	Utility evaluation of the MRD-guided therapy
Lung	LUN0114 (NCT04585490)	U.S	Interventional, phase III	48	AVENIO ctDNA Surveillance Kit	Stage III, NSCLC, post-CRT, on consolidation therapy	Utility evaluation of the MRD-guided therapy
Lung	MERMAID-1 (NCT04385368)	Global	Interventional, phase III	89	ArcherDx^™^	Stage II–III, NSCLC, scheduled resection	MRD( +): RCT of durvalumab + chemotherapy vs. chemotherapy
Lung	MERMAID-2 (NCT04642469)	Global	Interventional, phase III	30	ArcherDx^™^	Stage II–III, NSCLC, post-curative treatment	MRD( +): RCT of durvalumab vs. placebo
Lung	MRD-LUNG (NCT06111807)	Germany	Observational	248	NA	Stage III, NSCLC, scheduled curative treatment	MRD monitoring
Lung	JCOG2111A	Japan	Observational	350	Signatera^™^	Stage IB–III, NSCLC, scheduled curative treatment	MRD monitoring
Breast	Safe-De (NCT05058183)	U.K	Observational	400	Signatera^™^	HER2 + , TNBC, Stage I	MRD monitoring
Breast	HARMONY (NCT05433753)	Japan	Observational	60	Todai OncoPanel	HER2 + , Stage II–III	MRD monitoring
Breast	JCOG1204A1	Japan	Observational	248	Signatera^™^	Stage II–III	MRD monitoring
Breast	LEADER (NCT03285412)	U.S	Interventional, phase II	120	Signatera^™^	HR + HER2-, T1c–T4c, postoperative, MRD positive	RCT of ribociclib + endocrine therapy vs. endocrine therapy
Breast	DARE (NCT04567420)	U.S	Interventional, phase II	100	Signatera^™^	HR + HER2–, Stage II–III, on adjuvant chemotherapy, MRD positive	RCT of palbociclib + fulvestrant vs. continuation of adjuvant chemotherapy
Breast	TRAK-ER (NCT04985266)	EU	Interventional, phase II	1100	Invitae PCM^™^	HR + HER2–, on adjuvant chemotherapy, MRD positive	RCT of palbociclib + fulvestrant vs. continuation of adjuvant chemotherapy
Breast	NCT05388149	Canada	Interventional, phase II	15	RaDaR^™^	HER2 + , Stage I–III, on adjuvant chemotherapy, MRD positive	Efficacy evaluation of neratinib + trastuzumab emtansine
Breast	PERSEVERE (NCT04849364)	U.S	Interventional, phase II	197	FoundationOne Liquid CDx^™^	TNBC, Stage I–III, NAC + postoperative	Efficacy evaluation of the MRD and genome-guided therapy
Breast	ASPRIA (NCT04434040)	U.S	Interventional, phase II	40	NA (Sysmex Inostics)	TNBC、NAC + postoperative, MRD positive	Efficacy evaluation of atezolizumab + sacituzumab govitecan
Bladder	IMVigor011 (NCT04660344)	Global	Interventional, phase III	495	Signatera^™^	pT2-4a or N + , postoperative, MRD positive	RCT of atezolizumab vs. placebo
Bladder	TOMBOLA (NCT04138628)	Denmark	Interventional, phase II	282	Tumor-informed	cT2-4a, postoperative, MRD positive	Atezolizumab
Renal	KIDNEY-PAGER (NCT06145139)	Denmark	Observational	500	NA	Scheduled resection	MRD monitoring
Liver	COSMOS-HCC (UMIN000041710)	Japan	Observational	200	Guardant Reveal^™^	Scheduled resection/RFA	MRD monitoring
Liver	REMNANT (NCT05375370)	France	Observational	150	NA	Scheduled resection/RFA	MRD monitoring
Liver	NCT06157060	China	Observational	255	NA	Scheduled resection	MRD monitoring
Liver	ctDNA-HCC-01 (NCT05823584)	Taiwan	Observational	207	Tumor-naïve (Vh-DNA)	HBV + , scheduled resection	MRD monitoring
Liver	NCT06382103	China	Observational	120	NA	Scheduled resection	MRD monitoring
Biliary tract	COSMOS-BTC (UMIN000052780)	Japan	Observational	110	Guardant Reveal^™^	Scheduled resection	MRD monitoring
Biliary tract	NCT06171321	China	Interventional	94	NA	Stage II–III, Scheduled resection	Efficacy evaluation of the MRD-guided therapy
Pancreatic	ARTEMIS-PC	Japan	Observational	50	PCM^™^	Scheduled resection	MRD monitoring
Pancreatic	COSMOS-PC	Japan	Observational	500	Guardant Reveal^™^	Scheduled resection	MRD monitoring
Pancreatic	MAP-02 (NCT05802407)	China	Interventional	100	NA	On adjuvant chemotherapy, MRD positive	RCT of treatment modification vs. continuation of current treatment
Pancreatic	MAP-03 (NCT05802394)	China	Interventional	100	NA	BR/LA, on chemotherapy, MRD positive	RCT of treatment modification vs. continuation of current treatment
Gastrointestinal	MRD-GI (NCT05482516)	U.S	Interventional, phase II	20	Signatera^™^	Post-curative treatment, MRD positive	Efficacy evaluation of atezolizumab + bevacizumab
Ovarian	Nir-Bev (jRCT2031220732)	Japan	Randomized, phase II	70	Signatera^™^	Stage III–IV, post-debulking surgery, MRD positive	RCT of niraparib vs. niraparib + bevacizumab
Ovarian	NCT06341907	China	Interventional, Phase II/III	10	NA	Stage II–IV, MRD positive	Efficacy evaluation of neoantigen polypeptide vaccine
Ovarian	NCT05931055	China	Observational	1000	Fragmentomics	Scheduled resection	MRD monitoring
Endometrial	NCT06341855	China	Interventional	100	NA	Postoperative, high recurrence risk	MRD( +): RCT of treatment vs. follow-up MRD(-): Follow-up
Cervical	NCT06283875	China	Observational	80	NA	Stage IB–IVA, scheduled resection/CRT	MRD monitoring
Cervical	NCT05950087	China	Observational	30	NA	Scheduled CRT	MRD monitoring
Cervical	NCT05872724	China	Interventional Phase II	32	NA	Stage IB2–II2, postoperative	Efficacy evaluation of MRD-guided therapy
Cervical	NCT05531981	China	Observational	350	Tumor-naïve (HPV E7)	Stage IA2–IVA	MRD monitoring
Pharyngeal	NRG-HN001 (NCT02135042)	U.S	Interventional, phase II/III	685	Tumor-naïve (EBV-DNA)	Nasopharyngeal cancer, post-RT	MRD( +): RCT of PF therapy vs. GT therapy MRD(-): RCT of PF therapy vs. follow-up
Pharyngeal	T1313 (NCT02363400)	Taiwan	Interventional, phase III	147	Tumor-naïve (EBV-DNA)	Nasopharyngeal cancer, post-RT, MRD positive	RCT of MEP therapy vs. follow-up
Pharyngeal	NCT05307939	U.S	Interventional, phase II	30	NavDx^®^	HPV + , oropharyngeal cancer, post-operative, MRD positive	Arm A: Delay or omission of RT Arm B: Shortening of CRT period
Pharyngeal	SAVAL (NCT06088381)	U.S	Interventional, phase II	61	Tumor-naïve (HPV-DNA)	HPV + , oropharyngeal cancer, scheduled resection	Efficacy evaluation of treatment according to the risk and MRD status
Pharyngeal	NCT06445114	U.S	Interventional, phase II	50	NavDx^®^	HPV + , oropharyngeal cancer, scheduled resection/post-operative	Efficacy evaluation of radiation dose adjustment according to the risk and MRD status
Pharyngeal	SPHERE (NCT04965792)	U.S	Observational	150	NavDx^®^	HPV + , oropharyngeal cancer, post-curative treatment	MRD monitoring
Pharyngeal	SURVEILLE-HPV (NCT05582122)	France	Randomized, phase II	420	ddPCR, Tumor-naïve (HPV-DNA)	HPV + , oropharyngeal cancer, post-curative treatment	MRD monitoring
Head and neck	NeckTAR (NCT05710679)	France	Interventional	63	Tumor-informed	SCC, Stage III–IVB, scheduled RT	MRD monitoring
Skin	NCT05736523	U.S	Observational	28	Signatera^™^	Melanoma, Stage II–III, scheduled resection	MRD monitoring
Skin	PERCIMEL (NCT04866680)	France	Observational	165	Tumor-informed	Melanoma, Stage III/IV, postoperative	MRD monitoring
Skin	Clear Me (NCT06319196)	Canada	Interventional, phase II	54	RaDaR^™^	Melanoma, Stage III/IV, postoperative, MRD positive	Nivolumab + relatlimab vs. nivolumab
Skin	NCT06246227	Denmark	Observational	467	NA	Melanoma, Stage IIB–IV, postoperative	MRD monitoring
Skin	COSMOS-MEL-01 (UMIN000042040)	Japan	Observational	50	Guardant Reveal^™^	Melanoma, Stage IIB–IV, scheduled resection	MRD monitoring
Skin	COSMOS-MEL-02 (UMIN000051210)	Japan	Observational	50	Guardant Reveal^™^	Melanoma, *BRAF* V600 + , Stage IIB–IV, postoperative	MRD monitoring
Multiple cancer	UMBRELLA (NCT06332274)	France	Interventional, phase III	717	NA (BeiGene, C2iGenomics)	Post-curative treatment	MRD( +): RCT of tislelizumab vs. placebo MRD(-): RCT of follow-up every 3 months vs. follow-up every 6 months
Multiple cancer	MARIA (NCT05219734)	U.S	Observational	400	PCM^™^	Patients with non-distant metastases	MRD monitoring
Multiple cancer	ORACLE (NCT05059444)	U.S	Observational	1000	Guardant Reveal^™^	Scheduled resection	MRD monitoring
Multiple cancer	CAMPERR (NCT05366881)	U.S	Observational	7000	NA	Before treatment initiation	MRD monitoring
Multiple cancer	SCRUM-Japan MONSTAR-SCREEN-3 (UMIN000053975)	Japan	Observational	3200	Precise MRD	Diagnosed with malignant solid tumor, and curative resection or curative chemotherapy, radiation therapy, or chemoradiotherapy is planned	MRD monitoring

*RCT* randomized controlled trial, MRD molecular residual disease, *NA* not available, MSS microsatellite stability, *TROP2-CAR-NK* trophoblast cell surface antigen 2-chimeric antigen receptor-natural killer cells, *TNT* total neoadjuvant therapy, *NAC* neoadjuvant chemotherapy, *TNBC* triple negative breast cancer, *HR* hormone receptor, *HER2* human epidermal receptor 2, *RT* radiation therapy, *CRT* chemoradiation therapy, *HBV* hepatitis B virus, *NSCLC* non-small cell lung cancer, *SCC*, squamous cell carcinoma, *GIST* gastrointestinal stromal tumor, *EGFR* epidermal growth factor receptor, *PF* cisplatin and fluorouracil, *GT* gemcitabine and paclitaxel, *MEP* mitomycin-C, epirubicin, cisplatin, oral tegafur-uracil

##### Colorectal cancer

Several randomized clinical studies based on MRD testing are currently ongoing in Japan. The VEGA study is a randomized phase III study that aims to verify the non-inferiority of follow-up observation compared with CAPOX adjuvant therapy in high-risk stage II/low-risk stage III postoperative MRD-negative patients. The ALTAIR study is a randomized phase III study verifying the superiority of FTD/TPI over placebo in MRD-positive patients under recurrence surveillance. The AURORA study is a randomized phase II study on verifying the superiority of mFOLFOXIRI plus bevacizumab therapy to mFOLFOX6 therapy in patients who were MRD-positive after surgery for oligometastases (screened in the COSMOS-CRC-03 study) [28, 29]. These studies are expected to have a significant impact on the direction of future clinical development of MRD testing. Nonetheless, MRD test results still require careful interpretation, as several important issues remain to be resolved, such as with regard to the accuracy of MRD assays and the appropriate selection of target patient populations. Attention should also be paid to how MRD testing using ctDNA analysis can be incorporated into colorectal cancer treatment in the future. To fully realize the benefits of precision medicine based on MRD testing, obtaining high-level evidence, therapeutic development strategies, and efforts are required, with a view to its use in clinical practice.

##### Gastric cancer

Several randomized studies are being conducted on postoperative MRD testing in gastric cancer, although all of them are small-scale studies (*N* = 46–80 patients each), and no large-scale studies have been initiated to date. Nevertheless, several prospective observational studies have been conducted and have demonstrated the utility of MRD testing and the predictive ability of ctDNA analysis in response to neoadjuvant chemotherapy.

It has been noted that for recurrence in the form of peritoneal dissemination, which is characteristic of gastric cancer, the detection sensitivity may be reduced due to low ctDNA levels [30]. To resolve this issue, it is desirable to develop more sensitive assays and establish an MRD testing method that can complement sensitivity. In the future, large-scale prospective studies are expected to help verify the clinical validity of MRD testing and to promote the development of precision medicine approaches.

##### Esophageal cancer

In addition to neoadjuvant CRT, curative-intent treatment with CRT is the standard of care for esophageal cancer. Research focusing on the utility of MRD testing during and after curative intent treatment is also underway, mainly in Asian countries. In Japan, the DISCOVER study (jRCT1071240001) is being conducted to evaluate ctDNA-positive status before, during, and after surgery using Guardant Reveal. There are three groups (*N* = 200 patients) in this study: preoperative untreated, neoadjuvant chemotherapy, and neoadjuvant CRT groups. The results of the study need to be interpreted with caution because of the complexity of the treatment strategy; however, it could demonstrate the clinical validity of MRD testing in esophageal cancer, further promoting the development of relevant precision medicine strategies.

### Lung cancer

#### Status of evidence accumulation

A PubMed search of the literature, excluding case reports, using the keywords circulating tumor DNA (or ctDNA, minimally residual disease, molecular residual disease), surgery (or neoadjuvant, chemoradiation), and lung cancer yielded 685 reports published as of May 2024. After narrowing them down to those relevant to MRD testing after curative-intent treatment in patients with lung cancer, 41 reports remained; specifically, six retrospective studies, 30 prospective observational studies, and five meta-analyses, with no randomized studies reported to date.

#### Major literature reports (Table 1)

The prospective observational TRACERx study by Abbosh et al. found that MRD positivity was significantly associated with poor prognosis in patients with stage I–III non-small cell lung cancer in the UK. [31]. In this study, tumor-informed ctDNA analysis was performed on 197 patients who underwent surgery using probes targeting a median of 200 (range 72–201) tumor-specific genomic alterations per patient, which were divided into MRD-positive and MRD-negative groups based on their status within 120 days of surgery and before the start of adjuvant therapy, to compare their prognosis. The MRD positivity rate within 120 days of surgery was 25%, indicating a significantly higher risk of recurrence in the MRD-positive group than in the MRD-negative group (HR: 6.8 [95% CI 3.7–12.3]). In addition, the median time from MRD detection to clinical recurrence during recurrence surveillance (lead time) was 119 days (range 0–1137 days). It should be noted that the preoperative ctDNA positivity rate was reported to be higher for histological cancer types other than adenocarcinoma than for adenocarcinoma.

In 2023, Chen et al. reported similar results in their prospective observational study of 181 patients with stage I–III non-small cell lung cancer who underwent surgery in China. In this study, tumor-informed ctDNA analysis targeted up to 50 tumor-specific genomic alterations per patient. The HR for MRD-positive recurrence-free survival at 30 days after surgery was 8.86 (95% CI 3.7–21.1), and the median lead time from MRD detection to diagnosis of clinical recurrence was 299 days [32].

In their meta-analysis of literature published till April 2022 (14 articles, *N* = 1051 patients), Chen et al. found that the HR for the risk of recurrence in postoperative MRD-positive patients was 6.52 (95% CI 5.08–8.36) [33].

In contrast, in the randomized IMpower 010 study comparing atezolizumab and best supportive care in patients who underwent cisplatin-based adjuvant chemotherapy after curative-intent resection for stage IB–IIIA non-small cell lung cancer, atezolizumab treatment prolonged disease-free survival (HR: 0.66 [95% CI 0.50–0.88]) [34] in the primary analysis population (stage II–IIIA patients with PD-L1 expression 1% or higher). MRD testing using Signatera was performed as an exploratory analysis of the IMpower 010 study data and revealed that the postoperative MRD positivity rate increased with the advancement of the disease stage (stage IB: 9%; stage II: 14%; and stage IIIA: 29%) and that the HR for MRD-negative stage II–IIIA patients relative to that for MRD-positive patients was 0.72 (95% CI 0.52–1.00) in the atezolizumab group and 0.61 (95% CI 0.39–0.94) in the best supportive care group. Therefore, MRD positivity is consistently a poor prognostic factor [35]. In the overall primary analysis population, disease-free survival was prolonged regardless of postoperative MRD status (HR for MRD-positive patients: 0.54 [95% CI 0.31–0.93]; HR for MRD-negative patients: 0.57 [95% CI 0.36–0.90]). Disease-free survival was prolonged in MRD-negative patients with PD-L1 expression ≥ 50% (HR: 0.35 [95% CI 0.16–0.75]) but not in those with PD-L1 expression 1–49% (HR: 0.78 [95% CI 0.43–1.42]). This suggests that treatment de-escalation may be used in some patients, depending on the MRD status [36].

In addition, the utility of MRD testing has been reported for recurrence surveillance after curative-intent CRT. Pan et al. performed MRD testing after curative-intent CRT on 139 patients, mainly with unresectable locally advanced clinical stage III non-small cell lung cancer. Regarding progression-free survival, the HR for the risk during progression surveillance in MRD-negative patients was 0.18 (95% CI 0.12–0.28) [37]. Similarly, many other studies have reported the utility of MRD testing for recurrence surveillance after curative-intent treatment.

Several observational studies have analyzed the efficacy of adjuvant therapy in association with postoperative MRD positivity. Shen et al. conducted a meta-analysis covering reports published up to May 2022 and showed that the HR for the efficacy of adjuvant therapy in MRD-positive patients was 0.27 (95% CI 0.17–0.44). In contrast, in MRD-negative patients, the HR was 1.51 (95% CI: 0.81–2.79), which did not confirm the efficacy of adjuvant therapy [38].

#### Expected clinical development in the future (Table 2)

Several randomized clinical studies based on postoperative MRD testing are currently underway. Although no data from randomized studies are currently available for lung cancer, published observational and retrospective studies have consistently reported the utility of MRD testing for recurrence prediction. While awaiting the results of randomized studies, the utility of MRD testing is expected for future clinical development of precision onco-surgery.

### Breast cancer

#### Status of evidence accumulation

A PubMed search using the keywords for breast cancer, ctDNA, and operable (or early) yielded 651 reports published as of May 2024. Reviews and meta-analyses were excluded, after which the search was further narrowed to studies conducted within the past 10 years using the keywords retrospective, observational, and clinical trials, which yielded 18, 10, and 42 reports, respectively. Thereafter, combined with hand-searched literature, reports considered essential were reviewed.

#### Major literature reports (Table 1)

Nader-Marta et al. conducted a meta-analysis to evaluate the associations between preoperative ctDNA positivity/postoperative MRD positivity and disease-free and overall survival in patients with stage I–III breast cancer (3174 reports, 57 studies; *N* = 5779 patients) [39]. MRD-positive patients under recurrence surveillance had a poor prognosis for disease-free survival (HR, 14.04; 95% CI 7.55–26.11); patients who tested positive for ctDNA after neoadjuvant chemotherapy also had a poor prognosis (HR, 7.69; 95% CI 4.83–12.24). For overall survival, recurrence surveillance had an HR of 9.19 (95% CI 3.26–25.90), and after neoadjuvant chemotherapy, the HR was 2.72 (95% CI 1.44–5.14); similar results were obtained using multivariate analysis. The reported lead time was 10.81 months (range 0–58.9 months). Furthermore, the association between ctDNA positivity, disease-free survival, and overall survival was particularly higher in the tumor-informed assay than in the tumor-naive assay.

In a multicenter, randomized phase II c-TRAK TN interventional study, patients who underwent triple-negative breast cancer surgery followed by MRD testing using digital PCR every 3 months for 12 months were compared to those in the intervention group, in which patients with no clinical recurrence at the time of MRD-positive conversion were administered pembrolizumab and to the follow-up observation group. The primary endpoints were MRD detection and MRD-negative conversion rates during pembrolizumab treatment. The MRD positivity rate was 27.3% (44 of 161 patients, 95% CI 20.6–34.9) at 12 months. Seven patients experienced recurrence while remaining MRD-negative, and in 72% of patients in the intervention group, clinical recurrence was observed when MRD-positive conversion was identified. Five patients in the intervention group received pembrolizumab, but none of them continuously maintained MRD-negative status. Recurrence had already been confirmed by imaging in many cases by the time of MRD-positive conversion. Therefore, the results highlight several issues that need to be addressed and suggest the importance of performing MRD testing frequently from the early postoperative period onwards, using an assay with higher sensitivity [40].

Several similar reports have been published on the risk of recurrence in MRD-positive patients involving either ctDNA analysis after neoadjuvant chemotherapy or MRD testing after surgery and during recurrence surveillance. Data for all studies were reported according to subtype, and the risk of recurrence was consistently high in patients with preoperative ctDNA positivity or postoperative MRD positivity, irrespective of the setting and subgroup.

#### Expected future clinical development (Table 2)

Several clinical trials are being conducted to evaluate MRD over time during recurrence surveillance and whether new therapeutic interventions performed at the time of MRD-positive conversion can improve prognosis. Based on the results of these studies, the development of more personalized precision medicine strategies that focus on patients with poor prognoses is expected in the future. One representative clinical study is the LEADER study, and some of its results were reported at the San Antonio Breast Cancer Symposium in 2023. This study enrolled 191 patients with hormone receptor-positive, HER2-negative breast cancer. At the median follow-up time point of 12 months, 10.1% (17 patients) of 168 patients who were eligible for ctDNA analysis using Signatera were MRD-positive. Among these 17 MRD-positive patients, the percentage of patients with no recurrence on imaging or clinically at the time of MRD-positive conversion (true molecular relapse) was 70.6% (12 of 17 patients) [41]. Future results are expected to determine whether the addition of CDK4/6 inhibitors improves the prognosis of these patients. Issues to be addressed when conducting such clinical trials include the low MRD positivity rate of 10%, which makes the number of patients insufficient to enable randomization. Approximately 30% of the patients already had clinical relapse by the time of MRD-positive conversion, lack of information regarding the appropriate MRD testing interval and the appropriate drugs to be used in the intervention group.

### Urologic cancer

#### Status of evidence accumulation

A PubMed search using the keywords prostate cancer, bladder cancer, and renal cell carcinoma, in combination with circulating tumor DNA and surgery for each of them, yielded eight, 20, and 11 reports, respectively, as of June 2024.

#### Major literature reports (Table 1)

##### Prostate cancer

Recurrence surveillance for prostate cancer is being conducted using the tumor-specific marker prostate-specific antigen; therefore, there have been a relatively limited number of reports on MRD testing in prostate cancer. However, some studies have reported that the detection of ctDNA before treatment affects the recurrence-free survival of patients undergoing total prostatectomy for localized prostate cancer. Pope et al. performed tumor-informed preoperative ctDNA analysis using whole genome sequencing (INVAR) to increase sequencing depth further. They showed that both biochemical recurrence-free survival (HR: 3.3 [95% CI 1.4–8.1]) and recurrence-free survival (HR: 2.8 [95% CI 1.1–7.1]) were significantly shorter in preoperative ctDNA-positive patients than in ctDNA-negative patients [42]. There are also other reports on preoperative ctDNA analysis with targeted sequencing focusing on some genes.

##### Urothelial cancer

It has been reported that more ctDNA is released in urothelial cancer than in other cancer types [43]. Therefore, evidence of MRD is most abundant in the context of the urinary system. Bladder cancer accounts for 90% of all urothelial cancers and has been studied using targeted sequencing with ddPCR. In recent years, multiple clinical studies using Signatera, a tumor-informed assay, have been conducted. Christensen et al. performed MRD testing using Signatera before and after curative-intent cystectomy. They reported that the postoperative MRD positivity rate was 26.6%, the recurrence rate in postoperative MRD-positive patients was 76%, the sensitivity and specificity for recurrence were extremely high (100% and 98%, respectively), and the median lead time to clinical recurrence in MRD-positive patients under recurrence surveillance was 96 days [44]. In the IMvigor010 study, Powles et al. performed MRD testing using Signatera. They reported improvements in recurrence-free survival (HR: 0.58 [95% CI 0.43–0.79]) and overall survival (HR: 0.59 [95% CI 0.41–0.86]) in the atezolizumab group as compared with those in the follow-up group of postoperative MRD-positive patients (37%) [45]. However, there are only two reports on MRD for upper urinary tract urothelial cancer originating from the renal pelvis and ureter. Furthermore, Christensen et al. used ctDNA analysis of urine and plasma to consider the characteristics of bladder cancer [46]. Specifically, they performed ddPCR of driver genes (*FGFR3* and *PIK3CA*) and reported significantly lower recurrence-free survival duration in urinary ctDNA-positive patients. A small-scale study on upper urinary tract urothelial cancer, which occurs at a low incidence, also used postoperative ddPCR and target-sequencing-based MRD analysis.

##### Renal cancer

Renal cancer is known to be a low ctDNA-shedding type, along with head and neck cancer and melanoma. The number of reports related to MRD is limited, and there are some reports on perioperative methylation analysis of specific genes, considering the low levels of ctDNA. Buttner et al. reported significantly shortened recurrence-free survival (HR: 5.89 [95% CI 1.46–23.8]) in the group with high *SHOX2* promoter methylation frequency, which is reported to be associated with prognosis in other cancer types [47].

#### Expected future clinical development (Table 2)

##### Prostate cancer

As of June 2024, no studies have aimed at detecting postoperative MRD. However, if the MRD detection rate improves with the advancement of sequencing technology, it is expected to promote further investigations, such as whether perioperative ctDNA/MRD status is associated with recurrence-free survival rate and other clinical prognosis indicators independent of PSA.

##### Urothelial cancer

Based on the results of the abovementioned IMvigor010 study, the IMvigor011 study is being conducted to evaluate the efficacy of atezolizumab as an adjuvant therapy for MRD-positive patients with muscle layer-invasive bladder cancer. This study is expected to determine the utility of adjuvant therapy depending on the presence or absence of postoperative MRD.

##### Renal cancer

In August 2022, pembrolizumab was included in the national health insurance reimbursement coverage as the first adjuvant therapy for renal cell carcinoma. Therefore, MRD testing may be required to identify the groups that truly benefit from adjuvant therapy, as in the IMvigor011 study on urothelial cancer. Newer technologies, such as whole-genome sequencing and cfDNA methylation analysis, are expected to improve the MRD detection rate in the future [48].

### Hepatobiliary and pancreatic cancer

#### Status of evidence accumulation

A PubMed literature search using the keywords molecular residual disease, circulating tumor DNA, and surgery yielded 369 reports published until February 2024. After combining the results of a subsequent manual search, 412 reports were reviewed. Hepatocellular carcinoma, biliary tract cancer, and pancreatic cancer were reported in the hepatobiliary and pancreatic regions (11, 5, and 21 cases, respectively).

#### Major literature reports (Table 1)

##### Pancreatic cancer

In the field of pancreatic cancer, which has the largest number of reports, most studies have used MRD testing based on ctDNA analysis of *KRAS* mutations. Lee et al. performed ctDNA analysis of *KRAS* mutations before and after resectable pancreatic cancer surgery. They reported that the preoperative ctDNA positivity rate was 62.2%, and the postoperative MRD positivity rate was 37.1% [49]. Preoperative ctDNA-positive patients had worse recurrence-free survival (HR: 4.1 [95% CI 1.8–9.0]) and overall survival (HR: 4.1 [95% CI 1.6–10.5]) than ctDNA-negative patients. The overall survival of the positive patients was comparable to that of the unresected patients. In addition, postoperative ctDNA-positive patients had poor recurrence-free survival (HR: 5.4 [95% CI 1.9–15.2]) and overall survival (postoperative, HR: 4.0 [95% CI 1.2–13.6]). According to a meta-analysis of *KRAS*-mutant MRD-positive patients who underwent pancreatectomy, the prognosis was poor. However, the HR tended to be lower than that for other cancer types (HR: 3.32 [95% CI 2.19–5.03]) [50]. Lee et al. conducted an interventional study (the DYNAMIC-Pancreas study, which used tumor-informed ctDNA analysis for MRD testing) in postoperative pancreatic cancer patients, where adjuvant chemotherapy was administered for 6 months in the MRD-positive group and for 3–4 months in the MRD-negative group. They reported that the prognosis was significantly better in the latter (recurrence-free survival: 13 vs 22 months, HR: 0.28). However, as the median recurrence-free survival of 22 months was still poor, they concluded that reduction of the adjuvant therapy period for MRD-negative patients should not be recommended [51]. In Japan, neoadjuvant chemotherapy is adopted as the standard of care; however, its impact on the interpretation of postoperative MRD results has not been clarified and will require further investigation.

##### Hepatocellular carcinoma

Patients with postoperative MRD-positive hepatocellular carcinoma patients have also been reported to have a poor prognosis. Ye et al. used ctDNA for MRD testing within 7 days of surgery. They reported that MRD-positive patients (*N* = 23 of 96, 24.0%) who underwent resection for hepatocellular carcinoma had a significantly poor prognosis in terms of recurrence-free survival (HR: 6.074 [95% CI 2.648–13.929]) and overall survival (HR: 4.829 [95% CI 1.508–15.466]) [52]. Furthermore, MRD positivity was revealed to be an independent prognostic factor for previously identified risk factors such as high alpha-fetoprotein (AFP) levels, microscopic vascular invasion, and Barcelona Clinic Liver Cancer (BCLC) stage.

##### Biliary tract cancer

Patients with postoperative MRD-positive biliary tract cancer have also been reported to have a poor prognosis, although the number of reports is limited. King et al. reported a significantly poor recurrence-free survival (HR: 7.4 [95% CI 2.6–4758]) in a study on ctDNA analysis of 12 perioperative MRD-positive biliary tract cancers (*N* = 3/9, 33%) [53].

#### Expected future clinical development (Table 2)

##### Pancreatic cancer

The prognosis of pancreatic cancer is extremely poor, and effective treatment options are limited. Therefore, MRD testing is expected to be an effective technique for facilitating multidisciplinary treatment strategies for this cancer [54]. However, at present, most reports on MRD testing for pancreatic cancer use ctDNA analysis for *KRAS* mutations, raising the issue of low sensitivity. The development of highly sensitive assay methods, such as a tumor-naive methylation-based ctDNA detection assay and a tumor-informed assay based on whole-exome and whole-genome analyses, is in progress [55–57]. Regarding the development of perioperative treatments based on ctDNA testing, many studies have reported that the postoperative overall survival in preoperative ctDNA-positive patients is very poor, comparable to that in patients with distant metastases. Therefore, the use of ctDNA testing to determine eligibility for surgery or neoadjuvant therapy and for efficacy assessment has been considered [1, 5, 58, 59]. Regarding the postoperative period, as the DYNAMIC-Pancreas study concluded that reduction in the duration of adjuvant therapy is not recommended for MRD-negative patients, treatment intensification for MRD-positive patients should be discussed rather than treatment simplification for MRD-negative patients [3]. A randomized clinical study (NCT05802407) is ongoing in China to verify the efficacy of modified adjuvant chemotherapy in MRD-positive patients under recurrence surveillance [60].

##### Hepatocellular carcinoma

Several large-scale prospective studies are ongoing to verify the clinical validity of MRD testing in hepatocellular carcinoma [57] [61–65]. Current clinical studies on adjuvant therapy, in comparison with follow-up observational groups, include the IMbrave050 study on atezolizumab plus bevacizumab, the CheckMate9DX study on nivolumab, the KEYNOTE-937 study on pembrolizumab, and the EMERALD-2 study on durvalumab plus bevacizumab. The IMbrave050 study on patients at high risk of recurrence showed no significant difference in recurrence-free survival in the subgroup with a tumor diameter ≤ 5 cm (HR: 1.06 [95% CI 0.65–1.74]) [66]. For patients who undergo surgery for hepatocellular carcinoma, identifying those at high risk of recurrence is considered to be an issue, and MRD testing may be useful in this regard. In addition, as radiofrequency ablation is the standard of care for patients with hepatocellular carcinoma, treatment development based on MRD testing in patients who have undergone curative-intent non-operative treatment is also expected.

##### Biliary tract cancer

Similar to pancreatic cancer, the prognosis of biliary tract cancer is poor, and effective treatment options are limited. Therefore, MRD testing is expected to be an effective technique to establish multidisciplinary treatment strategies. Furthermore, prospective studies assessing the clinical validity of MRD testing are also in progress. However, the number of these studies is very limited [57] [67, 68], and there have been no interventional studies on perioperative treatments based on MRD testing. Therefore, future prospective studies are expected to help evaluate the clinical validity of MRD testing, potentially leading to advancements in treatment development.

### Gynecological cancer

#### Status of evidence accumulation

A PubMed literature search using the keywords ovarian cancer, cervical cancer, and uterine cancer in combination with circulating tumor DNA for each of them yielded 143, 92, and 50 articles, respectively, published till May 2024. However, reports on MRD testing are limited, and there are even fewer prospective studies (three studies on ovarian cancer, one on endometrial cancer, and three on cervical cancer). Notably, there has been only one confirmatory study on cervical cancer.

#### Major literature reports (Table 1)

##### Ovarian cancer

The reported preoperative ctDNA positivity rate in patients with ovarian cancer ranges from 58.6% to 93%. Heo et al. performed ctDNA analysis using a tumor-naive assay for nine genes in patients who underwent debulking surgery for ovarian cancer (*N* = 22) and reported a preoperative ctDNA positivity rate of 69.2% [69]. Patients who were ctDNA-positive before treatment initiation and 6 months later had worse progression-free survival (HR: 10.7 [95% CI 4.4–25.9]) than those who were ctDNA-negative before treatment initiation and those with negative ctDNA conversion 6 months later. In contrast, Hou et al. performed perioperative ctDNA analysis in stage I–IV patients using Signatera. They reported a preoperative ctDNA positivity rate of 73% and a postoperative MRD positivity rate of 33% [70]. In patients eligible for recurrence surveillance based on MRD testing, both the sensitivity and specificity for recurrence in MRD-positive patients were 100%, with a mean lead time from MRD positivity to clinical recurrence of 10 months. At the end of curative treatment, MRD-positive patients had significantly worse recurrence-free survival than MRD-negative patients (HR: 17.6 [95% CI 3.2–97.4]). Kallio et al. performed perioperative ctDNA analysis in stage I–IV patients using a tumor-informed assay. The ctDNA positivity rate before treatment initiation was 93% [71]; furthermore, at the time of the final testing during the treatment period, the MRD-positive patients had significantly worse progression-free survival (HR: 5.63) and overall survival (HR: 8.22) than the MRD-negative patients.

##### Endometrial cancer

Ashley et al. performed ctDNA analysis in stage I–IV patients using a tumor-naive assay of 129 genes and reported that the preoperative ctDNA positivity rate was 22%, and the postoperative MRD positivity rate was only 6.7% [72]. Both preoperative ctDNA-positive patients (HR: 11.14 [95% CI 2.72–45.59]) and postoperative MRD-positive patients (HR: 15.56 [95% CI 2.16–112.16]) had significantly worse progression-free survival.

##### Cervical cancer

Both surgery and radiation therapy are used as curative treatments for cervical cancer. Jeannot et al. used ddPCR of the E7 gene of human papillomavirus (HPV) for ctDNA analysis. The positivity rate was 63% before treatment [73]; at the end of treatment, MRD-positive patients had significantly shorter progression-free survival than MRD-negative patients (HR: 10.95 [95% CI 2.94–40.7]). The mean lead time to clinical recurrence in the MRD-positive group under recurrence surveillance was 10 months. Han et al. conducted a prospective study on CRT in patients with stage IB–IVA cervical cancer [74] and reported HPV ctDNA positivity before treatment in 70 (93.3%) of 75 patients. Three of the five patients who tested negative underwent cervical HPV screening and were HPV-negative. The progression-free survival of MRD-positive patients after treatment was significantly shorter than that of MRD-negative patients (HR, 8.58 [95% CI 3.56–20.71]). In addition, analysis using next-generation HPV sequencing instead of ddPCR showed significantly shorter progression-free survival in MRD-positive patients after treatment than in MRD-negative patients (HR: 4.19 [95% CI 1.76–9.98]).

#### Expected clinical development in the future (Table 2)

##### Ovarian cancer

The GALAXY-OV study (UMIN000050754), a prospective, observational study evaluating the efficacy of MRD testing using the tumor-informed Signatera assay in advanced ovarian cancer, is currently ongoing. In addition, the Nir-Bev study (jRCT2031220732), a randomized phase II study comparing niraparib maintenance therapy and niraparib plus bevacizumab combination maintenance therapy in MRD-positive patients enrolled in the GALAXY-OV study after neoadjuvant chemotherapy plus interval debulking surgery (IDS), is currently ongoing. These interventional studies are expected to clarify the utility of MRD status as a prognostic factor in advanced ovarian cancer, the natural history of MRD-positive patients during adjuvant chemotherapy and maintenance therapy, and the utility of bevacizumab added to niraparib maintenance therapy in post-IDS-MRD-positive-patients who-are-considered to be at high risk of recurrence. Other ongoing studies include observational MRD monitoring studies and a study in which a neoantigen polypeptide vaccine was administered to patients who underwent ovarian cancer surgery and whose MRD status is being monitored (NCT06341907).

##### Endometrial cancer

A study (NCT06341855) comparing follow-up and additional treatment in postoperative MRD-positive patients was initiated in China.

##### Cervical cancer

Ongoing studies include an observational MRD monitoring study and a study in which adjuvant treatment consisting of CRT plus anti-PD-1 antibody therapy with or without chemotherapy was administered after surgery (NCT05872724).

### Head and neck cancer

#### Status of evidence accumulation

A PubMed search for literature published till June 2024 using the keywords circulating tumor DNA and head and neck cancer yielded 72 reports, whereas a search with the keywords head and neck cancer, HPV, and plasma yielded 53 reports, and a search with the keywords nasopharyngeal carcinoma, EBV (Epstein–Barr virus), and plasma yielded 399 reports.

#### Major literature reports (Table 1)

##### Nasopharyngeal cancer

Numerous studies have been conducted on MRD testing through the detection of EBV-DNA in the blood using quantitative PCR and other methods. Lin et al. assessed the significance of EBV-DNA in the blood as a prognostic factor in patients with locally advanced nasopharyngeal cancer who underwent curative-intent radiation therapy and reported that the prognosis of MRD-positive patients was significantly worse (2-year recurrence-free survival: 28.6% vs 84.2%) than that of MRD-negative patients [75]. Chen et al. conducted a study on recurrence surveillance using blood EBV–DNA in patients who had completed radiation therapy. They reported that the sensitivity and specificity for recurrence were 82.3% and 80.0%, respectively, with a median lead time of 2.3 months [76]. Peng et al. conducted a meta-analysis and reported that the sensitivity and specificity of blood EBV DNA detection for recurrence were 85.8% and 89.0%, respectively [77].

The significance of personalization of nasopharyngeal cancer treatment based on MRD testing has not yet been established. The NPC 0502 study, a randomized phase III study including 104 patients who were MRD-positive after curative CRT, compared the MRD-guided group (six cycles of adjuvant chemotherapy with gemcitabine plus cisplatin) and the non-adjuvant chemotherapy and follow-up groups and found no difference between the groups in the primary endpoint of the 5-year recurrence-free survival rate (49.3% vs 54.7%, HR: 1.98 [95% CI 0.63–1.89]) [78]. We speculate that the reason for the absence of a significant difference was the adjuvant chemotherapy regimen used in the MRD-guided group.

##### HPV-related oropharyngeal cancer

There are reports from Japan and other countries on MRD testing using qPCR for detecting high-risk HPV DNA in the blood. Chera et al. used blood HPV DNA detection for recurrence surveillance after CRT and reported a median lead time of 6.6 months. The sensitivity and specificity for recurrence, by defining two consecutive MRD-positive results as recurrence, were 100% for both, and MRD-positive patients had a significantly worse prognosis than MRD-negative patients (2-year recurrence-free survival: 50% vs 100%) [79]. Hanna et al. used NavDx^®^, which detects tumor-derived HPV-DNA, and reported that the sensitivity and specificity for recurrence in MRD-positive patients were 87.3% and 99.4%, respectively [80]. Jensen et al. conducted a meta-analysis and reported that the sensitivity and specificity for recurrence determined based on blood HBV-DNA in MRD-positive patients were 54% and 98%, respectively [81]. Regarding the reasons for the low sensitivity in this meta-analysis, the authors considered that there were issues regarding tumor volume, blood collection, and storage methods.

The significance of MRD testing for personalized treatment of HPV-associated oropharyngeal cancer has not yet been established. A phase II study (NCT05307939) on the omission, delay, or reduction of adjuvant radiation therapy in postoperative MRD-negative patients is currently in progress. However, the cohort in which the initiation of adjuvant radiation therapy was delayed or omitted until MRD-positive conversion in the population with pathologically intermediate risk factors was terminated prematurely due to a failure to demonstrate the utility of MRD testing [82].

##### HPV-unrelated head and neck squamous cell carcinoma

Several attempts have been made to use ctDNA analysis to identify somatic genomic alterations in patients with HPV-unrelated head and neck squamous cell carcinoma. Honore et al. evaluated the utility of an NGS-based tumor-naive assay (with 26 genes, including two HPV genes) in locally advanced cancer (*N* = 53; 17 HPV-related and 36 HPV-unrelated) and reported that the prognosis of MRD-positive patients was significantly worse than that of MRD-negative patients (2-year progression-free survival, 23.53% vs 86.6%) [83]. Flach et al. evaluated the utility of RaDaR in cases of resection for HPV-unrelated head and neck squamous cell carcinoma (*N* = 17). They reported that clinical recurrence occurred in all six MRD-positive patients, with a lead time of 108 to 253 days [84].

#### Expected future clinical development (Table 2)

##### Nasopharyngeal cancer

The personalization of adjuvant chemotherapy based on MRD testing after curative-intent radiation therapy has been considered, and several clinical studies are ongoing. Of these, a particularly important one is the NRG-HN001 study, in which adjuvant chemotherapy with cisplatin plus 5-FU and adjuvant chemotherapy with gemcitabine plus paclitaxel were compared in the MRD-positive patients who underwent curative-intent radiation therapy and also adjuvant chemotherapy with cisplatin plus 5-FU and follow-up observation were compared in MRD-negative patients.

##### HPV-related oropharyngeal cancer

Several prospective interventional studies on the personalization of adjuvant chemotherapy based on MRD testing and recurrence surveillance using MRD testing after postoperative radiation therapy are ongoing. The NCT05307939 study enrolled patients who were pathologically high-risk and underwent radiation therapy after debulking surgery. The phase II SURVEILLE-HPV study is being conducted to compare two groups—one in which patients undergo follow-up observation at a conventional visiting frequency and the other in which MRD testing is introduced while visiting frequency is reduced and in the case of positive conversion MRD testing is performed every two months and magnetic resonance imaging (MRI) with positron emission tomography/computed tomography (PET/CT) is performed every four months.

##### HPV-unrelated head and neck squamous cell carcinoma

For locally advanced head and neck squamous cell carcinoma, both HPV-related and HPV-unrelated, the ongoing NeckTAR study aims to compare MRD testing using a tumor-informed assay in the presence or absence of residual lesions based on PET/CT. Further evidence regarding the significance of MRD testing in HPV-unrelated head and neck squamous cell carcinoma and the development of novel MRD-based treatments are expected in the future.

### Skin cancer

#### Status of evidence accumulation

A PubMed search for literature published until September 2024 using the keywords circulating tumor DNA, MRD, melanoma, non-melanoma skin cancer, squamous cell carcinoma, basal cell carcinoma, extramammary Paget disease, cutaneous adnexal carcinoma, and Merkel cell carcinoma in addition to manual literature search for references in review articles on the same theme yielded 10 reports for melanoma and one report for non-melanoma skin cancer. Literature focusing on circulating tumor DNA in uveal melanoma was excluded because its primary treatment differs from other melanoma subtypes.

#### Major literature reports (Table 1)

##### Melanoma

All available reports were observational studies. Most studies first confirmed the presence of driver mutations, such as *BRAF* and *NRAS*, in tumor tissues and then tested for these mutations in blood samples using ddPCR [85–90]. Other methods used in these studies included BEAMing in one study [91], real-time PCR in one study [92], and the tumor-informed Signatera assay in two studies [93, 94]. Tan et al. used ddPCR for MRD testing of postoperative patients with stage III melanoma with a *BRAF*, *NRAS*, *TERT*, *TP53*, or *KIT* mutation (*N* = 133 patients). The MRD positivity rates before and after surgery were 35% and 24%, respectively; with regard to the risk assessment of recurrence-free survival, the HR was 2.9 (95% CI 1.5–5.6) for preoperative ctDNA-positive patients and 10 (95% CI 4.3–24) for postoperative MRD-positive patients [87]. Eroglu et al. used Signatera for MRD testing of 69 patients with melanoma (30 postoperative patients at stage III and 39 patients with unresectable disease or distant metastasis). The postoperative MRD positivity rate in the stage III group was 17%, and the HR for distant metastasis-free survival in MRD-positive patients relative to MRD-negative patients was 10.77 (95% CI 1.77–65.57) [94].

In observational studies that evaluated MRD (*N* = 10), the postoperative MRD positivity rate was generally around 20% to 30%, although there were considerable variations between studies, possibly due to differences in sample sizes, stages, assessment time points, and assays used. Although the HR for recurrence-free survival in MRD-positive patients ranged from 2 to 10, it was generally correlated with poor prognosis. The sensitivity for recurrence also ranged from approximately 10% to 80% but tended to be low in general. In contrast, the specificity was approximately 80% to 100%, tending to be high. Therefore, the development of assays with superior detection sensitivity is required.

##### Non-melanoma skin cancer

Non-melanoma skin cancers include cutaneous squamous cell carcinoma, basal cell carcinoma, extramammary Paget disease, cutaneous adnexal carcinoma, and Merkel cell carcinoma. In an observational study of Merkel cell carcinoma, Akaike et al. used the Signatera assay for MRD testing (*N* = 319 patients with stage I–III Merkel cell carcinoma). Among the 84 patients who became clinically negative for the disease after curative-intent surgery or radiation therapy, the 1-year recurrence-free survival rate was 26% for MRD-positive patients and 79% for MRD-negative patients (HR, 7.4; 95% CI 2.7–20.3) [95].

#### Expected future clinical development (Table 2)

##### Melanoma

Most of the ongoing prospective studies are observational studies using the MRD positivity rate as an endpoint. Clinical studies verifying treatment strategies to intensify or simplify perioperative treatment based on the MRD status of patients with curatively resectable melanoma are expected. The DETECTION study (NCT04901988) was a randomized phase II/III study to compare recurrence surveillance in stage IIB/C melanoma with *BRAF*, *NRAS*, or *TERT* promoter mutations between the standard group (in which follow-up observation was performed until recurrence without regard for MRD test results) and the MRD-guided group (in which MRD-positive patients received nivolumab) [96]. However, the DETECTION study was prematurely terminated because surgery alone was no longer a standard of care since adjuvant anti-PD-1 became available for stage IIB/C melanoma in 2021. The ClearMe study (NCT06319196) is a phase II study of postoperative stage III/IV melanoma with MRD clearance as the endpoint. In this study, MRD testing was performed using the tumor-informed RaDaR assay, and MRD-positive patients were randomized to either adjuvant nivolumab plus relatlimab combination therapy or adjuvant nivolumab therapy [97]. It is anticipated that the establishment of a high-sensitivity assay will lead to an increase in clinical studies verifying MRD-based treatment strategies in the future.

##### Non-melanoma skin cancer

Perioperative systemic therapy has not been approved for non-melanoma skin cancers. Therefore, no ongoing interventional studies have verified MRD-guided perioperative treatment strategies. If observational studies demonstrate the utility of MRD testing, as in the case of Merkel cell carcinoma, it is expected that clinical studies will be conducted in the future to verify MRD-guided treatment strategies.

## Clinical questions (CQs)

The Position Paper on Appropriate Clinical Use of MRD Testing comprehensively covers resectable solid tumors and is an aggregation of expert opinions on the respective tumor types. Although there may be conflicting or weak evidence for some tumor types, we prioritized consensus building in developing the recommendations to share the utility of MRD testing with patients. Therefore, although there may be cases where the details of the recommendation may require different interpretations depending on the tumor type, we hope that these recommendations will be interpreted appropriately with reference to the guidelines for the respective tumor types before being used clinically.

### Determination of the recommendation level

In the development of this guideline, CQs were established to address clinically relevant questions, and evidence that provides the rationale for answers to these CQs was collected by manual search. Based on these results, 15 Working Group members voted to determine the recommendation level for each CQ (Table 3). Voting was conducted based on the strength of the evidence for each CQ, including the expected benefits and losses for the patients. Regulatory approval and insurance reimbursement status in Japan regarding medical treatments (including indications for examinations and treatment) were not considered in the voting process and are noted as remarks in the commentaries, as needed.

**Table 3:** Recommendation level determined by voting and assessment criteria

Recommendation level	Criteria for assessing the recommendation level	Description
Strong recommendation (SR)	Strongly recommended, because evidence is sufficient and benefits outweigh losses	Strongly recommended
Recommendation (R)	Recommended taking into account the balance of benefits and losses, because a certain level of evidence exists	Recommended
Expert consensus opinion (ECO)	Evidence and information on benefits is insufficient, but a certain level of consensus has been obtained	Consideration is recommended
No recommendation (NR)	Not recommended, because of no evidence	Not recommended

The results of the recommendation level for each CQ were determined by voting as follows:

1. SR (Strongly recommended) if SR votes were 70% or more,
2. R (Recommended) if (1) was not met and SR + R votes were 70% or more,
3. ECO (Expert consensus opinion) if (1) and (2) were not met and SR + R + ECO votes were 70% or more,
4. NR (Not recommended) if NR votes were 50% or more, regardless of (1) to (3), and.
5. N/A (Recommendation level not applicable) if none of (1) to (4) were met.

CQ1: Which assays are recommended for postoperative MRD testing? *Recommendation 1–1*: Assays that have demonstrated analytical and clinical validity for MRD testing are strongly recommended.

*Recommendation level*: Strongly recommended [SR: 15, R: 0, ECO: 0, NR: 0]

*Recommendation 1–2*: The use of MRD assays that have demonstrated higher clinical validity in postoperative recurrence risk evaluation than auxiliary examinations for recurrence diagnosis, such as those using tumor markers and diagnostic imaging, is strongly recommended.

*Recommendation level*: Strongly recommended [SR: 14, R: 1, ECO: 0, NR: 0]

*Recommendation 1–3*: The use of MRD assays that have demonstrated higher clinical validity in recurrence surveillance than auxiliary examinations for recurrence diagnosis, such as those using tumor markers and imaging tests, is strongly recommended.

*Recommendation level*: Strongly recommended [SR: 12, R: 3, ECO: 0, NR: 0]

**Commentary**

MRD testing identifies recurrence at the molecular level ("molecular recurrence") before the evidence of clinical, biological, or radiological recurrence (hereafter referred to as "recurrence"). MRD testing for solid tumors primarily involves ctDNA analysis.

The performance of the laboratory test is evaluated from three perspectives: analytical validity, clinical validity, and clinical utility. The "Guidelines for Genetic Testing and Diagnosis in Medical Care" (Japanese Association of Medical Sciences, February 2011, revised in March 2022) define each of these as follows [98].

- Analytical validity refers to ensuring that the test method has been established and appropriate precision controls are implemented to obtain highly reproducible results. It is evaluated based on information such as the positivity rate when a pathological variant (mutation) is present, the negativity rate when a pathological variant (mutation) is absent, the presence or absence of a quality control program, and procedures of confirmatory tests.
- Clinical validity refers to the ability to ensure that the interpretation of the test results is fully determined and evaluated based on information such as sensitivity (positivity rate when the disease is present), specificity (negativity rate when the disease is absent), morbidity rate, positive predictive value, negative predictive value, and genotype–phenotype relationship.
- Clinical utility refers to the feature of delivering clinical benefits such as promoting the understanding and acceptance of the disease by the patient and their family members by providing a diagnosis of the target disease, obtaining information that can improve the prospects of the patient, and leading the patient to appropriate prevention or treatment methods. It is evaluated based on information, such as the impact of test results on the patient and whether effective treatment options are available.

The performance of each characteristic of the MRD testing can be evaluated using the following parameters:

- Analytical validity: Accuracy, sensitivity, precision, and cross-reactivity of MRD detection.
- Clinical validity: The relationship between recurrence and the MRD test results is evaluated using clinical specimens. Specifically, it is evaluated using the following indices, with recurrence as the control:
  •Sensitivity (percentage of MRD-positive patients among those who had a recurrence)
  •Specificity (percentage of MRD-negative patients among those who had no recurrence)
  •Positive predictive value (percentage of MRD-positive patients who had a recurrence)
  •Negative predictive value (percentage of MRD-negative patients who had no recurrence)
  •HRs for overall survival, disease-free survival, and recurrence-free survival in MRD-positive patients relative to those in MRD-negative patients
  •Duration from MRD detection to recurrence in recurrence surveillance (lead time)
- Clinical utility: Impact of therapeutic and other interventions based on MRD testing results on clinical outcomes

To ensure the reliability of the MRD testing results, the establishment of analytical validity and the implementation of appropriate precision control that provides highly reproducible results is essential. In addition, whether MRD testing has detected molecular recurrence prior to recurrence should be evaluated based on clinical validity using clinical specimens. In fact, the Medicare and Medicaid Programs in the US also require established clinical validity as a prerequisite for insurance reimbursement for an MRD assay [99]. Direct proof of its clinical utility requires long-term follow-up and large-scale clinical studies, which are time- and resource-intensive. However, as indicated for CQ2 to CQ9, there is a certain rationality based on the results of past clinical studies for the clinical use of MRD testing. Taken together, the recommendation level for an assay that has demonstrated analytical and clinical validity has been determined as "Strongly recommended." (Recommendation 1–1).

The objectives of MRD testing are classified into two categories.

1. "Postoperative recurrence risk assessment" to assist in determining adjuvant chemotherapy (CQ3, CQ6, and CQ8)
2. "Recurrence surveillance" to identify molecular recurrence prior to recurrence (CQ3 and CQ7)

Although all previous clinical studies were observational studies and not interventional studies, they have reported that patients who were MRD-positive in the postoperative recurrence risk assessment consistently showed a higher association with recurrence than those assessed based on tumor markers recommended in the recurrence surveillance for each solid cancer (Table 4). Similarly, although there were only a few reported cases in recurrence surveillance, MRD testing had a higher association with recurrence or detection earlier prior to recurrence compared with analysis based on the tumor markers recommended in recurrence surveillance for each solid tumor (Table 5). A meta-analysis comparing the clinical validity of carcinoembryonic antigen (CEA)-based analysis, MRD testing, PET, and CT for recurrence surveillance for colorectal cancer showed that sensitivity (CEA: 52%; MRD testing: 68%; PET: 95%; and CT: 77%) was the highest for PET, whereas specificity (CEA, 88%; MRD testing, 95%; PET, 87%; CT, 84%) and positive predictive value (CEA: 4.13, MRD testing: 12.83, PET: 7.15, CT: 4.78) were the highest for MRD testing [100]. For tumor types for which tumor marker-based auxiliary examinations are not recommended for recurrence diagnosis, the clinical validity of MRD testing should be evaluated in comparison with other standard methods of recurrence surveillance. Furthermore, in recurrence surveillance, it is desirable to set a longer lead time than in conventional recurrence surveillance.

**Table 4:** Clinical validity of tumor markers and MRD testing at the time of postoperative recurrence risk assessment in major clinical studies

Tumor type	Sample size	Association between MRD and recurrence	Association between tumor markers and recurrence	References
Colorectal cancer	96	HR (RFS): 3.8 (95% CI 2.4–21.0)	HR (RFS): 3.4 (95% CI 1.5–50)	[15]
Colorectal cancer	64	Sensitivity: 55.6% Specificity: 100%	Sensitivity: 35.0% Specificity: 80.7%	[16]
Colorectal cancer	299	HR (RFS): 17.0 (95% CI 8.7–34.0)	HR (RFS): 7.5 (95% CI 3.9–14.0)	[17]
Colorectal cancer	248	Sensitivity: 72%	Sensitivity: 44%	[101]
Hepatocellular carcinoma	50	HR (for recurrence): 5.79 (95% CI 2.41–20.52)	HR (for recurrence): 6.82 (95% CI 2.52–18.40)	[102]
Hepatocellular carcinoma	66	HR (RFS): 11.77 (95% CI 4.96–27.96)	HR (RFS): 4.77 (95% CI 2.08–10.97)	[103]
Pancreatic cancer	104	HR (DFS): 3.55 (95% CI 0.90–13.89)	HR (DFS): 2.69 (95% CI 0.57–12.75)	[104]
Pancreatic cancer	27	HR (OS): 5.02 (95% CI 1.23–7.66)	HR (OS): 2.14 (95% CI 0.60–7.66)	[105]
Pancreatic cancer	66	HR (DFS): 2.11 (95% CI 1.04–4.28)	HR (DFS): 1.96 (95% CI 0.76–5.04)	[106]
Ovarian cancer	12	HR (RFS): 7.34 (95% CI 0.75–72.3)	HR (RFS): 0.45 (95% CI 0.06–3.4)	[70]
Ovarian cancer	29	HR (PFS): 5.34 (95% CI 1.87–15.27)	HR (PFS): 1.12 (95% CI 0.15–8.58)	[107]

**Table 5:** Clinical validity of tumor markers and MRD testing in recurrence surveillance in major clinical studies

Tumor type	Sample size	Association between MRD and recurrence	Association between tumor markers and recurrence	References
Colorectal cancer	230	Sensitivity: 85% Median lead time: 167 days	Sensitivity: 41% Median lead time: 61 days	[14]
Colorectal cancer	48	Sensitivity: 53.3% Specificity: 100%	Sensitivity: 20% Specificity: 90.9%	[108]
Pancreatic cancer	25	HR (RFS): 34.95 (95% CI 5.19–235.4) HR (OS): 16.41 (95% CI 2.32–116.3)	HR (RFS): 3.01 (95% CI 1.18–7.67) HR (OS): 2.96 (95% CI 0.87–10.07)	[109]
Pancreatic cancer	39	HR (OS): 57.2 (95% CI 7.4–442.4)	HR (OS): 9.4 (95% CI 1.2–72.2)	[110]
Ovarian cancer	22	HR (RFS): *P* < 0.0001	HR (RFS): 4.3 (95% CI 0.96–19.5, *P* = 0.056)	[70]
Ovarian cancer	201	Median lead time: 2.3 months longer than that with CA125	–	[69]
Ovarian cancer	11	Median lead time: 49 days	Median lead time: 7 days	[111]

Taken together, the recommendation level for the use of an MRD assay that has demonstrated higher clinical validity in postoperative recurrence risk evaluation compared to auxiliary examinations for recurrence diagnosis, such as tumor markers and diagnostic imaging, has been determined as "Strongly recommended" (Recommendation 1–2). In addition, the recommendation level for the use of an MRD assay that has demonstrated higher clinical validity compared to auxiliary examinations for recurrence diagnosis for recurrence surveillance, such as tumor markers and diagnostic imaging, has also been determined as "Strongly recommended" (Recommendation 1–3).

CQ2: What patients are recommended for MRD testing? *Recommendation 2–1*: MRD testing for postoperative recurrence risk assessment is strongly recommended in patients who have undergone curative-intent resection.

*Recommendation level*: Strongly recommended [SR: 12, R: 3, ECO: 0, NR: 0]

*Recommendation 2–2*: MRD testing for recurrence surveillance is recommended for patients who have undergone resection with curative intent.

*Recommendation level*: Recommended [SR: 7, R: 8, ECO: 0, NR: 0]

**Commentary**

MRD testing identifies molecular recurrence prior to the manifestation of recurrence by performing minimally invasive ctDNA analysis in patients with cancer. Therefore, it has little significance for patients with confirmed evidence of recurrence or for those who have not undergone curative-intent resection. MRD testing should be performed in patients who have undergone curative-intent resections.

As shown in CQ1, there are two purposes for MRD testing after curative-intent resection: postoperative recurrence risk assessment and recurrence surveillance. For both purposes, the association between MRD positivity and recurrence has been reported in a tumor-type-agnostic manner (Tables 4 and 5). Therefore, the most significant use of MRD testing is for patients at a stage where the risk of postoperative recurrence is considered high according to the guidelines for each tumor type. However, in the GALAXY study, the postoperative MRD positivity rate in pathological stage I colorectal cancer, which is considered to have a low risk of recurrence, was low (0.9%). However, the HR for recurrence in MRD-positive patients was very high (35.51 [95% CI 6.99–180.35]) [20]. Therefore, even in stages with a low risk of recurrence, MRD testing for postoperative recurrence risk assessment is considered meaningful. Therefore, its implementation for patients who have undergone curative resection for postoperative recurrence risk assessment is determined as "Strongly recommended." However, regarding concrete tumor types and stages, it is considered a prerequisite that clinical validity be demonstrated in appropriate studies.

For recurrence surveillance, the opinion is that repeated implementation of MRD testing, which is quite costly, for patients who have been assessed as having a low recurrence risk is not always recommended from a medical economics perspective. In the GALAXY study, the 24-month disease-free survival rate was 95.6% in patients with pathological stage I colorectal cancer who were MRD-negative 4 weeks after surgery [20]. Therefore, for recurrence surveillance, MRD testing for patients who have undergone curative-intent resection is determined as "Recommended," which is not the highest level of recommendation, taking into account its significance for patients at low risk of recurrence. Regarding concrete tumor types and stages, it is also considered a prerequisite that clinical validity be demonstrated in appropriate studies.

CQ3: Which tests are recommended when MRD is detected during the postoperative recurrence risk assessment and surveillance? Recommendation: It is recommended to perform the tests for diagnosing recurrence that are recommended for each tumor type.

Recommendation level: Recommended [SR: 7, R: 4, ECO: 4, NR: 0]

**Commentary**

Guidelines for many tumor types recommend recurrence surveillance with the aim of early detection of recurrence. For instance, a meta-analysis conducted in Europe and the U.S. showed that postoperative surveillance contributed to an increased resection rate of recurrent tumors and improved the prognosis of colorectal cancer. Accordingly, guidelines recommend recurrence surveillance, including the use of tumor markers and CT [112]. For non-small cell lung cancer, one study reported that follow-up approaches did not improve postoperative prognosis. In contrast, others reported that intensive follow-up approaches improved survival rates and facilitated the treatment of other diseases. Therefore, regular follow-up with CT is recommended [113]. With regard to breast cancer, intensive surveillance for postoperative recurrence is not useful for patients at stages I–II, as they are at a low risk of recurrence. However, for patients with first-episode breast cancer at stage III or higher, improvement in treatment efficacy and quality of life is expected by detecting distant metastases and recurrence and initiating treatment when the tumor volume is low; therefore, recurrence surveillance incorporating various tests is permitted [114].

As indicated in the commentary on CQ1, a tumor type-agnostic association between MRD positivity and recurrence has been reported in both postoperative recurrence risk assessment and recurrence surveillance. As the reported lead time is 2–12 months, patients in whom MRD is detected may experience recurrence within several months. Although there is no evidence that early detection of recurrence in patients with detectable MRD improves prognosis, as noted above, early therapeutic intervention may lead to improvements in the resection rate, post-recurrence treatment efficacy, and quality of life, depending on the tumor type. There are some tumor types, such as breast cancer, for which there is no direct evidence of recurrence surveillance based on blood tests or imaging examinations, leading to an improvement in prognosis. Therefore, the necessity of diagnostic testing for recurrence when MRD is detected should be determined by tumor type. It is also unclear whether diagnostic testing for recurrence is necessary prior to adjuvant therapy if MRD is detected at the time of postoperative recurrence risk assessment. For these reasons, there was a wide variety of opinions from SR to ECO when determining the recommendation level for this CQ. Accordingly, while recognizing the potential benefits of early detection of recurrence, the overall recommendation level was determined as "Recommended," taking into consideration the characteristics of each tumor type and the limited evidence at present.

CQ4: When is the recommended time for implementing the MRD testing? Recommendation 4–1: For the purpose of postoperative recurrence risk assessment, implementation of MRD testing is recommended before the start of adjuvant therapy and at approximately 2–8 weeks after surgery.

Recommendation level: Recommended [SR: 9, R: 5, ECO: 1, NR: 0]

Recommendation 4–2: For the purpose of recurrence surveillance, consideration of implementation of MRD testing is recommended with a similar frequency and duration as those for recurrence surveillance for that tumor type.

Recommendation level: Consideration is recommended [SR: 0, R: 5, ECO: 6, NR: 4]

**Commentary**

The results of MRD testing for postoperative recurrence risk assessment may be used for decision-making regarding adjuvant therapy, considering the relevant tumor- and patient-related factors (CQ6 and CQ8). The optimal timing of adjuvant therapy is specified for each tumor type, and it is often recommended to start within 8–12 weeks of surgery. Since the turnaround time from blood collection for MRD testing to obtaining the results is 2–4 weeks [115, 116], testing should be performed around 4 weeks after surgery, or at the latest by 8 weeks after surgery, in order to use the results for decision-making regarding adjuvant therapy.

Notably, for approximately 4 weeks after the completion of surgery, normal cfDNA levels increase owing to the invasiveness of surgery, leading to false-negative results in the MRD testing [117]. A recent study reported that MRD can be detected 2–4 weeks after surgery, as in the period from 4 weeks after surgery onward [118]. Therefore, MRD testing should not be performed earlier than 2 weeks after surgery. Regarding the recommendation level, although "SR" votes were the majority, there were a certain number of "R" votes due to insufficient evidence. Therefore, for postoperative recurrence risk assessment, the overall recommendation level was determined as "Recommended" (Recommendation 4–1).

There is insufficient evidence regarding the optimal timing of recurrence surveillance using MRD testing for each tumor type. However, the guidelines recommend performing recurrence surveillance at a certain frequency and duration, depending on the recurrence pattern and risk of recurrence. Therefore, MRD testing for recurrence surveillance should also be performed according to the frequency and duration of recurrence surveillance recommended by each guideline.

In the CIRCULATE-Japan GALAXY study, 98% of patients with colorectal cancer and MRD-positive conversion showed conversion within 18 months of surgery [20]. Because a number of recurrences are observed 2–3 years after surgery for many tumor types, it is reasonable to consider performing surveillance using MRD testing during a specific period after surgery.

Several clinical studies evaluating the significance of therapeutic intervention for patients with MRD-positive conversion during the recurrence surveillance period are ongoing [28, 119]. Depending on the results of these studies, the frequency and duration of MRD testing during recurrence surveillance may vary.

Regarding the recommendation level, it was difficult to establish a uniform recommendation because of insufficient evidence and the fact that the frequency and duration of recurrence surveillance vary widely among tumor types. Therefore, the assessment of the recommendation level varied from R to NR, and the overall recommendation level was determined as "Consideration is recommended" (Recommendation 4–2). This recommendation level reflects the need to adopt MRD testing flexibly, depending on individual patients and clinical situations, while also recognizing its potential utility.

CQ5: Is preoperative ctDNA testing recommended for the prediction of recurrence? Recommendation: Consideration of implementation of preoperative ctDNA testing for the purpose of recurrence prediction is recommended.

Recommendation level: Consideration is recommended [SR: 0, R: 2, ECO: 12, NR: 1]

**Commentary**

There are no consistent results regarding the association between preoperative ctDNA detection and risk of recurrence. For instance, an analysis of 240 patients with stage II or III colorectal cancer using tumor-informed MRD testing showed that preoperative ctDNA-positive patients had significantly shorter recurrence-free survival than ctDNA-negative patients (HR: 5.66 [95% CI 1.72–18.57]) [120]. In contrast, the initial report of the CIRCULATE-Japan GALAXY study, which analyzed MRD status using Signatera in patients with stage II–IV colorectal cancer (*N* = 1039), showed no significant relationship between preoperative ctDNA and disease-free survival (HR: 0.89 [95% CI 0.55–1.4]) [121].

Studies reporting the clinical validity of both preoperative ctDNA and postoperative MRD have consistently shown that postoperative MRD is more strongly associated with the risk of recurrence than preoperative ctDNA (Table 6). However, it is also expected that preoperative ctDNA testing will be used in the future to evaluate the necessity of neoadjuvant treatment and determine its efficacy. Considering these potential benefits, the recommendation level was determined as "Consideration is recommended." However, further evidence is required for the use of preoperative ctDNA testing to evaluate the necessity of neoadjuvant treatment and to determine its efficacy.

**Table 6:** Clinical validity of preoperative ctDNA and postoperative MRD in major clinical studies and meta-analyses

Tumor type	Sample size	Association between preoperative ctDNA and recurrence	Association between postoperative MRD and recurrence	References
Colorectal cancer	1039	HR (DFS): 0.89 (95% CI 0.55–1.4)	HR (DFS): 10.0 (95% CI 7.7–14.0)	[20]
Colorectal cancer	299	Sensitivity: 94.4% Specificity: 25.2%	Sensitivity: 78.0% Specificity: 90.2%	[17]
Colorectal cancer	240	HR (RFS): 5.66 (95% CI 1.72–18.57)	HR (RFS): 10.98 (95% CI 5.31–22.72)	[120]
Non-small cell lung cancer	2143	HR (RFS): 3.00 (95% CI 2.12–4.24) HR (OS): 3.65 (95% CI 1.96–6.77)	HR (RFS): 4.95 (95% CI 3.06–8.02) HR (OS): 3.93 (95% CI 1.97–7.83)	[38]
Pancreatic cancer	868	HR (RFS): 2.07 (95% CI 1.35–3.17) HR (OS): 2.17 (95% CI 1.45–3.24)	HR (RFS): 3.32 (95% CI 2.19–5.03) HR (OS): 6.62 (95% CI 2.18–20.16)	[50]
Pancreatic cancer	104	HR (DFS): 0.65 (95% CI 0.20–12.75)	HR (DFS): 3.55 (95% CI 0.90–13.89)	[104]
Gastric cancer	50	HR (EFS): 3.0 (95% CI 1.3–6.9) HR (OS): 2.7 (95% CI 1.1–6.7)	HR (EFS): 21.8 (95% CI 3.9–123.1) HR (OS): 21.8 (95% CI 3.9–123.1)	[122]
Upper urinary tract urothelial cancer	43	HR (RFS): 5.69 (95% CI 1.83–17.71)	HR (RFS): 9.91 (95% CI 2.95–33.25)	[123]

CQ6: Is adjuvant therapy recommended for postoperative MRD-positive patients? Recommendation: Adjuvant therapy depending on each tumor type is recommended for postoperative MRD-positive patients.

Recommendation level: Recommended [SR: 7, R: 6, ECO: 2, NR: 0]

**Commentary**

It has consistently been reported that postoperative MRD-positive patients have a higher risk of recurrence than MRD-negative patients in a tumor type-agnostic manner (see Chapter 3). Adjuvant therapy has also been reported to improve the prognosis of postoperative MRD-positive patients. In the GALAXY study of patients with resectable colorectal cancer, high-risk pathological stage II and III patients who were MRD-positive after surgery and who underwent adjuvant chemotherapy had significantly longer disease-free survival than those without adjuvant chemotherapy (adjusted HR: 0.23 [95% CI 0.15–0.35], *P* < 0.0001) [20]. In addition, 68.8% of MRD-positive patients at 4 weeks after surgery had a negative conversion of MRD by 24 weeks after surgery due to adjuvant chemotherapy. In patients with MRD-negative conversion, compared with those without negative conversion, disease-free survival was significantly prolonged (HR for patients without adjuvant chemotherapy: 11.12 [95% CI 6.09–20.29], *P* < 0.0001). Negative conversion of postoperative MRD by adjuvant therapy has consistently been reported in colorectal cancer, although the proportion varies. Moreover, patients with negative conversion have consistently been reported to have a better prognosis than those without negative conversion [15] [124–126]. The IMvigor010 study, a randomized controlled study evaluating the efficacy of adjuvant atezolizumab therapy in patients with urothelial cancer who underwent surgery, reported that although the efficacy of atezolizumab was not observed in the entire study population (HR for recurrence-free survival: 0.89 [95% CI 0.74–1.08], *P* = 0.2446), atezolizumab significantly prolonged recurrence-free survival in postoperative MRD-positive patients, compared with those who underwent follow-up (HR: 0.58 [95% CI 0.43–0.79], *P* = 0.0024) [45, 127]. In patients who had MRD-negative conversion due to adjuvant atezolizumab therapy, compared with those without negative conversion, overall survival was significantly prolonged (HR: 0.14 [95% CI 0.03–0.59]). Furthermore, in a meta-analysis of patients with non-small cell lung cancer, adjuvant therapy for postoperative MRD-positive patients was also effective in terms of recurrence-free survival (HR, 0.27 [95% CI 0.17–0.44], *P* < 0.001) [38]. Therefore, similar results have been obtained for multiple tumor types regarding the efficacy of adjuvant therapy in postoperative MRD-positive patients. For patients with conditions for which the guidelines already recommend adjuvant therapy for each tumor type and who have postoperative MRD positivity, performing adjuvant therapy according to each tumor type is strongly recommended.

However, no studies have shown the efficacy of adjuvant therapy in patients with low recurrence risk, for whom the guidelines do not recommend adjuvant therapy for each tumor type. While adjuvant therapy is administered to many patients, including pancreatic cancer, lung cancer, and breast cancer, it is not recommended for non-advanced colorectal, gastric, head and neck, or renal cell cancers. In determining the recommendation level in this CQ, a divergence of views occurred among the Working Group members in respective regions, depending on the proportion of patients with low recurrence risk, for whom adjuvant therapy is not recommended by the guidelines for each tumor type, and assessment of the recommendation level varied from SR to ECO. Therefore, although the recommendation level was determined as "Recommended" with the objective of this guidance, which is to promote the appropriate clinical use of MRD testing in a tumor type-agnostic manner, we hope that the recommendation level will be interpreted in light of the significance of adjuvant therapy specified in the guidelines for each tumor type and used in clinical practice appropriately.

CQ7: Is treatment modification or additional treatment recommended for MRD-positive patients who are under surveillance for recurrence? Recommendation: As MRD-positive patients under recurrence surveillance are considered at a high risk of recurrence, consideration of treatment modification or additional treatment is recommended.

Recommendation level: Consideration is recommended [SR: 1, R: 2, ECO: 11, NR: 0]

**Commentary**

For several tumor types, it has been reported that the risk of recurrence is high in MRD-positive patients during surveillance. In the initial study of the GALAXY study of colorectal cancer, 32 of 692 MRD-negative patients at 4 weeks after surgery had MRD-positive conversion at 12 weeks after surgery, and the 18-month disease-free survival rate for patients with MRD-positive conversion was 33.8%, indicating a poor outcome [121]. In addition, 84 of 146 patients who were MRD-positive 4 weeks after surgery remained MRD-positive 12 weeks after surgery, and the 18-month disease-free survival rate for these patients was 22.9%, also indicating a poor outcome. A meta-analysis of patients with colorectal cancer who underwent resection revealed a higher risk of recurrence in MRD-positive patients at the end of adjuvant chemotherapy (HR: 10.59 [95% CI 5.59–20.06]) than in postoperative MRD-positive patients (HR: 7.27 [95% CI 5.49–9.62]) [128].

Similarly, in the IMVigor010 study on urothelial cancer, patients who were MRD-positive at the initiation of adjuvant atezolizumab therapy and remained MRD-positive until Day 1 of cycle 3 of therapy had a significantly worse prognosis than those who had negative conversion (HR for patients with MRD-negative conversion: RFS: 0.26 [95% CI 0.12–0.56], OS: 0.14 [95% CI 0.03–0.59]) [45]. In addition, in a meta-analysis of patients with lung cancer who underwent resection, no significant difference was observed in recurrence-free survival between patients who had MRD-positive conversion and those who remained MRD-positive after adjuvant chemotherapy (HR: 1.21 [95% CI 0.46–3.22]), showing a poor prognosis for both [38].

As described above, the risk of recurrence in MRD-positive patients under postoperative recurrence surveillance is very high for several tumor types, and several clinical studies are ongoing to verify the efficacy of treatment intensification in these patients. The c-TRAK TN study on postoperative triple-negative breast cancer was a randomized study that compared pembrolizumab with follow-up in recurrence-free patients who had MRD-positive conversion under recurrence surveillance [153]. In recurrence surveillance based on MRD conducted in 161 patients, 23 of 32 patients who had MRD-positive conversion were already confirmed to have recurrence at the time of positive conversion, and eventually, only five patients received pembrolizumab therapy. However, all five patients had recurrence, and no efficacy of treatment intensification for patients with MRD-positive conversion was observed. On the other hand, the ALTAIR study, a randomized study comparing FTD/TPI and placebo in disease-free patients with MRD-positivity under recurrence surveillance after curative-intent colorectal cancer treatment, has completed enrollment and is currently in the follow-up phase [154]. Therefore, MRD-positive patients under postoperative recurrence surveillance are at high risk of recurrence, and the efficacy of treatment modification or additional treatment is anticipated. However, currently, there are no available efficacy data. In determining the recommendation level for this CQ, ECO was the majority. Therefore, the recommendation level for treatment modification and additional treatment was determined as "Consideration is recommended." However, we agree that the recommendation level may be changed if the results of the ALTAIR study prove the efficacy of additional treatment in patients with MRD-positivity under surveillance.

CQ8: Is adjuvant therapy recommended for postoperative MRD-negative patients? *Recommendation*: For postoperative MRD-negative patients, consideration of adjuvant therapy according to tumor type is recommended. However, using less intense treatment regimens or not performing adjuvant therapy can also be a treatment option, taking into account the relevant tumor- and patient-related factors, etc.

*Recommendation level*: Consideration is recommended [SR: 3, R: 7, ECO: 5, NR: 0]

**Commentary**

The DYNAMIC study compared recurrence-free survival in postoperative patients with stage II colorectal cancer between a standard group, in which conventional clinicopathological factors were used as the eligibility criteria for adjuvant therapy, and the MRD-guided group, in which MRD testing results at 4 and 7 weeks after surgery were used as eligibility criteria. The 2-year recurrence-free survival rate was 93.5% in the MRD-guided group and 92.4% in the standard group (HR: 0.96 [95% CI 0.51–1.82], between-group difference: 1.1% [95% CI − 4.1%–6.2%], non-inferiority margin: − 8.5%). The proportion of patients receiving adjuvant chemotherapy was significantly lower in the MRD-guided group (15%) than in the standard group (28%), indicating non-inferiority of the MRD-guided group [19]. However, the 2-year recurrence-free survival rate in MRD-negative patients was significantly lower in clinicopathologically high-risk patients (89.7%) than in low-risk patients (97.4%) (HR: 3.04 [95% CI 1.26–7.34]), suggesting that conventional clinicopathological risk factors are also important for MRD-negative patients. In the GALAXY study, although no significant difference in disease-free survival was observed between MRD-negative patients at 4 weeks after surgery, irrespective of whether they underwent adjuvant therapy, the proportion of high-risk patients, such as those with lymph node metastases (82.2%) and those with pathological stage III (82.7%), was significantly higher in patients who underwent adjuvant therapy, further suggesting the appropriateness of performing adjuvant chemotherapy in consideration of clinicopathological risk factors even in MRD-negative patients [121 ]. The American Society of Clinical Oncology (ASCO) guidelines do not recommend uniform recommendation of adjuvant chemotherapy for pathological stage II patients but recommend its use in high-risk cases, such as those with T4 depth of invasion, vascular invasion, perforation, and tumor budding [129 ]. Currently, the VEGA study, a randomized study being conducted to assess the non-inferiority of follow-up to 3-month CAPOX therapy in postoperative MRD-negative patients with colorectal cancer, is ongoing, and its results are anticipated [28].

The DYNAMIC-Pancreas study, a prospective interventional study for resected pancreatic cancer, compared the recurrence-free survival of patients who were MRD-positive after surgery followed by 6 months of adjuvant chemotherapy and those who were MRD-negative after surgery followed by 3–4 months of adjuvant chemotherapy. The median recurrence-free survival was 22 months in the MRD-negative group and 13 months in the MRD-positive group, showing a significant difference (HR, 0.28; *P* = 0.003). However, the prognosis for MRD-negative patients was poor. Therefore, the study concluded that simplification of postoperative treatment for postoperative MRD-negative patients with pancreatic cancer was inappropriate [130]. Based on these results, evidence regarding safety of omitting or reducing the intensity of adjuvant therapy for postoperative MRD-negative patients is insufficient.

In contrast, several studies have reported that the prognosis of postoperative MRD-negative patients who were followed up without adjuvant treatment was better than that of MRD-positive patients who underwent adjuvant chemotherapy. In the GALAXY study on colorectal cancer, the 24-month disease-free survival rate of high-risk pathological stage II and III patients who were MRD-negative after surgery and followed up without adjuvant treatment was 89.9% (95% CI 86.80–92.30), which was better than that of postoperative MRD-positive patients who underwent adjuvant chemotherapy (35.83% [95% CI 27.41–44.32]) [20]. In the IMvigor010 study comparing the prognosis between adjuvant atezolizumab therapy and follow-up in patients with urothelial cancer, the median recurrence-free survival was assessed as "not yet reached" in postoperative MRD-negative patients who underwent follow-up without adjuvant treatment. At the same time, it was 5.9 months in MRD-positive patients who underwent adjuvant chemotherapy, showing a better result in postoperative MRD-negative patients who underwent follow-up without adjuvant treatment [45]. Similarly, in a prospective study in patients with non-small cell lung cancer, the median recurrence-free survival in postoperative MRD-negative patients who underwent follow-up without adjuvant treatment was assessed as "not yet reached." At the same time, it was 574 days in MRD-positive patients who underwent adjuvant chemotherapy, also showing a better result for postoperative MRD-negative patients who underwent follow-up without adjuvant treatment [131]. The results of the VEGA study provide further confirmation in this context. However, there have been no reports from observational studies that indicate a significant difference in the survival of MRD-negative patients in the presence and absence of adjuvant therapy. Therefore, for postoperative MRD-negative patients, there is a certain validity in the simplification of the standard treatment, considering the relevant tumor and patient factors. The use of less intense treatment regimens according to the guidelines for each tumor type and, in some cases, not performing adjuvant therapy can also be an alternative option.

Opinions varied widely from SR to ECO when determining the recommendation level for this CQ. Therefore, the overall recommendation level was determined as "Consideration is recommended."

CQ9: Is MRD testing recommended with curative-intent non-operative treatment? *Recommendation*: For patients undergoing curative-intent non-operative treatment, MRD testing is recommended for disease progression risk assessment and disease progression surveillance.

*Recommendation level*: Recommended [SR: 5, R: 7, ECO: 3, NR: 0]

**Commentary**

Curative-intent, non-operative treatment, which are already performed as part of the standard of care for solid tumors, include CRT and radiotherapy for lung, head and neck, esophageal, and cervical cancers; radiofrequency ablation therapy for hepatocellular carcinoma; and cryotherapy for renal cancer. In recent years, attempts have been made to achieve a radical cure as well as anal preservation using non-operative treatment involving immune checkpoint inhibitors for microsatellite instability-high rectal cancer. Biomarker-based curative-intent non-operative treatment for resectable solid tumors is also expected to be developed in the future. As the proportion of patients with solid tumors undergoing curative-intent non-operative treatment may increase, the significance of MRD testing for patients undergoing curative-intent non-operative treatment was further examined.

Moding et al. analyzed ctDNA in 65 patients with advanced non-small cell lung cancer before and after CRT and during consolidation therapy after CRT [132]. In the group receiving CRT alone, while all patients who were MRD-positive within 4 months of the end of CRT had disease progression within 12 months, all MRD-negative patients were progression-free (*P* = 0.006). In addition, while consolidation therapy significantly prolonged progression-free survival in patients who were MRD-positive after CRT (*P* = 0.04), it was not correlated with progression-free survival in MRD-negative patients (*P* = 0.23). These findings indicate that the presence or absence of MRD after CRT may be an eligibility criterion for consolidation therapy. Similarly, Pan et al. analyzed ctDNA in 139 patients with unresectable advanced non-small cell lung cancer before, during, and after CRT and during consolidation therapy. While consolidation therapy significantly prolonged progression-free survival in MRD-positive patients after CRT (HR: 0.54 [95% CI 0.32–0.89], *P* = 0.013), it did not correlate with progression-free survival in MRD-negative patients (HR: 0.69 [95% CI 0.35–1.37], *P* = 0.304) [37]. In addition, MRD-negative patients under progression surveillance (N = 28, 20.1%) had significantly longer progression-free survival than MRD-positive patients (HR: 0.18 [95% CI 0.12–0.28], *P* < 0.001), with a 2-year cancer-specific progression-free survival rate of 88.4%, suggesting the possibility of a curative effect. These studies demonstrated the utility of MRD testing in post-CRT disease progression risk assessment, disease progression surveillance, and personalized treatment strategies.

Similarly, the clinical validity of MRD testing in disease progression risk assessment and disease progression surveillance after curative-intent non-operative treatment has been reported for squamous cell carcinoma of the head and neck [79, 83], cervical cancer [73, 74], esophageal cancer [25, 133], and other cancers. Consistent results have been obtained for tumor types for which curative-intent non-operative treatment is the standard of care. Therefore, MRD testing for disease progression risk assessment and surveillance is "recommended” for patients undergoing curative-intent and non-operative treatment. Suggestions regarding the timing of testing are as follows, with reference to the recommendation for CQ4: for disease progression risk assessment at 2–8 weeks after the completion of curative-intent non-operative treatment, and for disease progression surveillance, based on the frequency and duration specified in the guidelines for each tumor type. Regarding treatment based on MRD testing results, although strategies similar to those recommended for patients undergoing curative-intent resection, as described for CQ6 to CQ8, can be considered, the evidence remains insufficient. Therefore, the validity of these treatments should be evaluated in future clinical studies.

In addition, most of the current evidence is for treatment after CRT or radiation therapy as curative-intent non-operative treatment. It is also considered appropriate to apply this recommendation to curative-intent non-operative treatments presented as part of the standard of care in the guidelines for each tumor type. We hope that this recommendation level will be interpreted in light of the significance of curative-intent non-operative treatment specified in the guidelines for each tumor type and will be used appropriately in clinical practice.

## Reference data

### Available MRD assays for solid tumors

In this position paper, we discussed the appropriate clinical use of MRD testing for solid tumors in a tumor type-agnostic manner. Various companies and academic institutions are working on the development of techniques for detecting MRD, some of which are commercially available in other countries as laboratory-developed tests (LDTs). The details of representative assays and their current availability (as of July 2024) are summarized in Tables 7 and 8, based on publicly available information from PubMed and the respective companies. However, assay names, names of the companies possessing the rights, and actual approval statuses may change with time. Furthermore, because information regarding the characteristics, performance, and indications of each assay is constantly being updated, it is important to confirm the latest information before actual clinical application. It should also be noted that the utility of MRD testing can differ depending on tumor type and stage. Therefore, each assay must be used after appropriate deliberation, depending on the target tumor type and clinical situation. Further technical innovation and accumulation of clinical evidence are expected to lead to the determination of the optimal assay for each case, thereby contributing to decision-making regarding treatment strategies.

**Table 7:** List of major tumor-informed MRD assays using personalized panels

Assay name	Development company/academic institution	Tumor tissue analysis method	Maximum number of target genes	U.S. CMS insurance reimbursement	U.S. FDA approval	U.S. marketing	CE marking	PMDA approval
Signatera^™^	Natera	WES	16	Colorectal cancer, bladder cancer, breast cancer, ovarian cancer	X	◯	◯	X
RaDaR^™^	NeoGenomics	WES	48	Breast cancer	X	◯	◯	X
PCM^™^	Invitae	WES	50	X	X	◯	X	X
StrataMRD^™^	Strata Oncology	WES	1–12	X	X	X	X	X
brPROPHET^™^	Burning Rock Dx	WES	50	X	X	X	X	X
Oncodetect^™^	Exact Sciences	WES	200	X	X	X	X	X
Haystack MRD^™^	Quest Diagnostics (Haystack Oncology)	WES	50	X	X	X	X	X
Foresight CLARITY^™^	Foresight Diagnostics	WGS	Unknown	X	X	◯	X	X
MRDetect	Veracyte	WGS	10,000	X	X	X	X	X
ppmSeq^™^	Ultima Genomics	WGS	10,000	X	X	◯	X	X
NEXT Personal^®^	Peronalis	WGS	1800	X	X	◯	X	X
MAESTRO	Broad Institute	WGS	10,000	X	X	X	X	X
Precise MRD	Myriad Genetics	WGS	1000	X	X	X	X	X

*CMS* Centers for Medicare & Medicaid Services, FDA Food and Drug Administration, CE marking Conformité Européenne marking, *PMDA* Pharmaceuticals and Medical Devices Agency, *WES* whole exome sequencing, *WGS* whole genome sequencing

**Table 8:** List of major tumor-naive MRD assays

Assay name	Development company/ academic institution	Analysis method (target of ctDNA analysis)	U.S. CMS insurance reimbursement	U.S. FDA approval	U.S. marketing	CE marking	PMDA approval
Guardant Reveal^™^	Guardant Health	DNA methylation	Colorectal cancer	X	◯	X	X
xM	Tempus	DNA methylation Gene mutation	X	X	◯	X	X
–	GRAIL	DNA methylation	X	X	X	X	X
NavDx^®^	Naveris	ddPCR	HPV-positive oropharyngeal cancer	X	◯	X	X
ColonAiQ^™^	Breakthrough Genomics	DNA methylation	X	X	◯	X	X

*ctDNA* circulating tumor DNA, *CMS* Centers for Medicare & Medicaid Services, *FDA* Food and Drug Administration, *CE Mark* Conformité Européenne Mark, *PMDA* Pharmaceuticals and Medical Devices Agency, *ddPCR* droplet digital PCR
